# Proportion, Function, and Generation of Dual Receptor Lymphocytes

**DOI:** 10.1155/jimr/5510858

**Published:** 2026-02-14

**Authors:** Qi Peng, Lanwei Zhu, XiaoPing Lu, Xinsheng Yao

**Affiliations:** ^1^ Department of Immunology, Center of Immunomolecular Engineering, Innovation and Practice Base for Graduate Students Education, Zunyi Medical University, Zunyi, China, zmc.edu.cn

**Keywords:** dual-BCR B cells, dual-receptor lymphocytes, dual-TCR T cells, receptor editing, single-cell RNA+VDJ sequencing technology, TCR^+^BCR^+^ lymphocytes, V(D)J allelic exclusion rearrangement, V(D)J allelic inclusion rearrangement

## Abstract

The specific immune response mechanisms of B cells and T cells are centered on the classic clonal selection theory, which posits that “a single lymphocyte expresses only one type of antigen receptor.” This mechanism is primarily achieved through V(D)J allelic exclusion rearrangement on germline chromosomes and rigorous self‐tolerance selection. However, accumulating experimental evidence indicates that phenomena such as incomplete allelic exclusion rearrangement (i.e., allelic inclusion rearrangement), escape from central tolerance selection, and peripheral immune receptor editing can induce the generation of dual‐receptor lymphocytes, including “dual‐BCR B cells,” “dual‐TCR T cells,” and “TCR^+^BCR^+^ lymphocytes.“ This article systematically reviews the research overview and recent advances in dual‐receptor lymphocytes in humans and mice under physiological and pathological conditions. By integrating theoretical model construction and validation results from immune molecular monitoring techniques, it emphasizes the proportional characteristics, biological effects, and possible origins of dual‐receptor lymphocytes. It also explores their association with disease development, aiming to provide a theoretical foundation and novel research perspectives for in‐depth studies in this field.

## 1. Introduction

The classical clonal selection theory proposing that “a single lymphocyte expresses only one type of antigen receptor” was introduced in the 1950s [1]. It has been confirmed that the primary mechanism underlying this phenomenon is allelic exclusion during germline V(D)J rearrangement: once a functional rearrangement of V(D)J genes occurs on one chromosome, feedback regulation and other mechanisms suppress the initiation of secondary recombination on the same chromosome as well as on the homologous chromosome. This process typically occurs in the T cell receptor (TCR) β chain and the B cell receptor (BCR) heavy chain; however, multiple rounds of allelic inclusion rearrangement can occur in the BCR κ (or λ) chain and the TCR α chain. Only during subsequent selftolerance selection does a single lymphocyte ultimately express only one type of light chain or α chain; lymphocytes that do not comply with rearrangement and selection rules undergo apoptosis [2–4]. To date, the core scientific questions regarding the regulatory mechanisms and molecules governing allelic exclusion and inclusion recombination, as well as the mechanisms and efficiency of escape from selftolerance selection, have not been fully elucidated due to the complexity of these processes [5–8].

However, as early as 1961, “dualBCR B cells” were first reported [9]; in 1988, “dualTCR T cells” were also first described [10–12]. Since then, various transgenic mouse models and specific T/B cell clones have become primary tools for studying the proportion and effects of dual receptor lymphocytes. Meanwhile, using specific antibodies targeting different TRBV families in humans and mice, as well as BCR κ and λ chains, followed by fluorescent labeling, dualTCR T cells and dualBCR B cells can be detected at the protein expression level by flow cytometry (FCM) and confocal fluorescence microscopy. Due to limitations in antibody specificity and coverage, reported proportions of dual‐receptor lymphocytes vary widely among different laboratories, ranging from 0.1% to 30%. At the same time, research on dual receptor lymphocytes heavily relies on techniques such as southern blot hybridization, Sanger sequencing, and single cell RNA sequencing (scRNAseq) to identify rearrangement characteristics at the mRNA or DNA level. In recent years, the rapid development of scRNAseq coupled with scV(D)Jseq technology has provided unprecedented technical support for studying dual receptor lymphocytes. The core advantage of this combined approach is its ability to analyze the pairing types and complementarity determining region 3 (CDR3) sequence features of BCR or TCR antigen receptor chains at high throughput single cell resolution, and to correlate these with the transcriptional expression profiles obtained from scRNAseq. This powerful tool has enabled indepth exploration of the proportion, characteristics, cellular origins, potential effects, and mechanisms of dual‐receptor lymphocytes, garnering widespread attention [13–19]. Furthermore, this technology has revealed the phenomenon of three or more types of singlechain mRNA expression in individual T or B cells, suggesting potential new mechanisms for the origin of dual‐receptor lymphocytes [18, 19]. In this review, based on the composition and rearrangement rules of VDJ genes in the murine IG loci (chromosomes 6, 12, and 16; Figure 1A) and TR loci (chromosomes 6 and 14; Figure 1B), we illustrate multiple VDJ recombination patterns of the IGH chain and TRB chain in the form of schematic diagrams, including the patterns of single functional rearrangement, dual functional rearrangement, multifunctional rearrangement, as well as the pairing mode of one (or more) functional IGH chain with one functional IG light chain, and that of one (or more) functional TRB chain with one functional TRA chain. Meanwhile, based on the specific sequence composition of individual cells derived from the shared single‐cell VDJ sequencing data (GSE140133; GSE168944), we list the family names of the corresponding VDJ sequence origins and the amino acid sequences of the CDR3 regions in single cells. Additionally, based on the composition and arrangement order of VDJC genes in the murine TRB locus, we constructed a schematic diagram detailing the sequential processes of VDJ recombination on a single mouse chromosome under the framework of classical VDJ recombination rules. This includes the inversion recombination involving the inverted V31 gene, the loop‐out recombination involving the forward Vx gene, as well as the canonical sequential order of D‐J rearrangement followed by V‐DJ rearrangement. Furthermore, we propose a potential mechanism by which two rounds of functional VDJ recombination and subsequent transcription can be accomplished on a single chromosome in accordance with classical recombination rules (Figure S1).

Figure 1Based on the composition and rearrangement rules of VDJ genes in the murine IG loci (chromosomes 6, 12, and 16; A) and TR loci (chromosomes 6 and 14; B), we illustrate multiple VDJ recombination patterns of the IGH chain and TRB chain in the form of schematic diagrams, including the patterns of single functional rearrangement, dual functional rearrangement, and multifunctional rearrangement, as well as the pairing mode of one functional IGH chain with one functional IG light chain and that of one functional TRB chain with one functional TRA chain. Meanwhile, based on the specific sequence composition of individual cells derived from the shared single‐cell VDJ sequencing data (GSE140133; GSE168944), we list the family names of the corresponding VDJ sequence origins and the amino acid sequences of the CDR3 regions in single cells.

**Figure:**
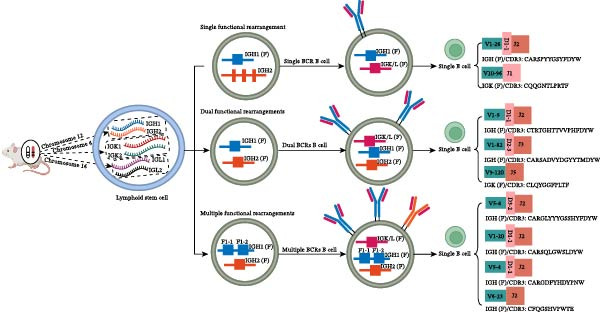
(A)

**Figure:**
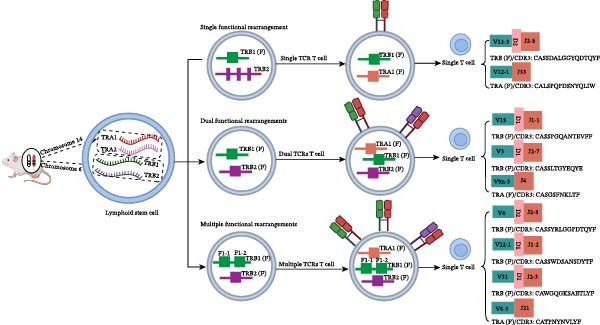
(B)

The continuous discovery and validation of dual‐receptor lymphocytes provide an important supplement to the classical clonal selection theory and the rules of V(D)J recombination. This article systematically reviews the research overview and progress on dualreceptor lymphocytes in humans and mice under physiological and pathological conditions since 1961, systematically summarizes the subjects, models, samples, methods, and techniques used in the literature, and outlines the types, proportions, characteristics, effects, regulatory mechanisms, and disease associations of dual‐receptor lymphocytes. In this review, in chronological order of publication, we classify the key literature on dual‐BCR B cells, dual‐TCR T cells, and TCR^+^BCR^+^ lymphocytes into two categories: in physiological conditions of humans and mice (Tables S1-1 and S2-1) and In pathological conditions of humans and mice (Tables S1-2 and S2-2: tumors, autoimmune diseases, infectious diseases, transplantation immunity, etc.). Meanwhile, we present the key findings from representative studies among them as illustrative results in accordance with the publication timeline (Figure 2), with a brief clarification of the objects, methods, and characteristics of dual‐TCR T cells, dual‐BCR B cells, and TCR^+^BCR^+^ lymphocytes in each study.

Figure 2Timeline of studies on the proportion, function, and generation mechanism of dual‐BCR B cells, dual‐TCR T cells, and TCR^+^BCR^+^ lymphocytes at key developmental nodes.

**Figure:**
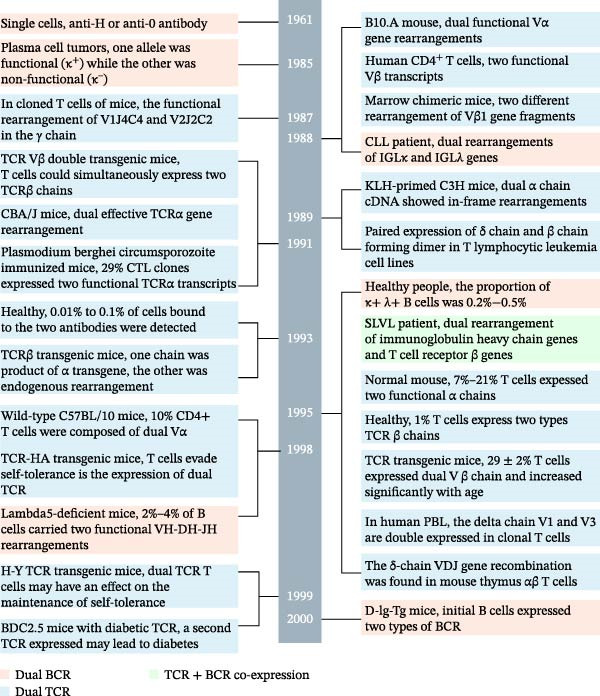

**Figure:**
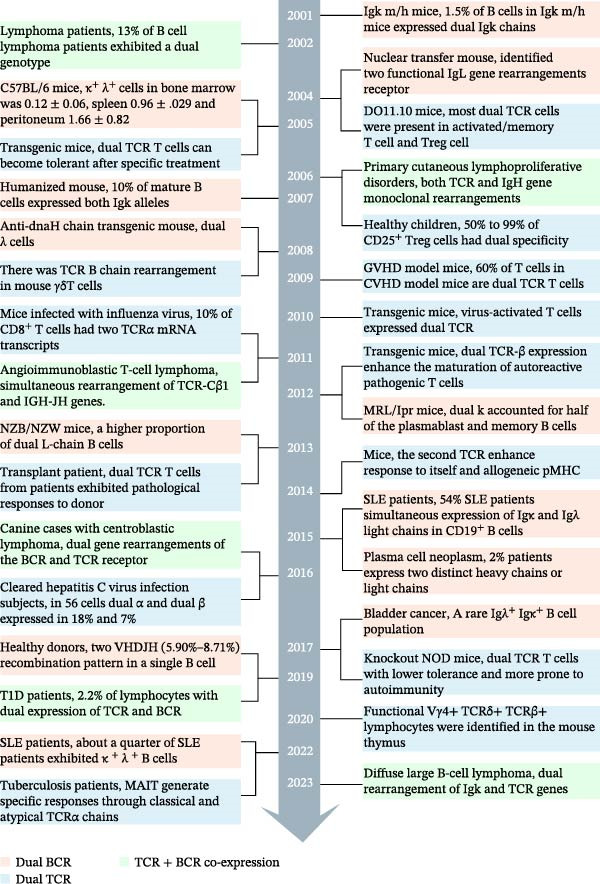

Meanwhile, we propose a systematic and standardized description for the general term “dual‐receptor lymphocytes” and its various subtypes: (1) General term: Dual‐receptor lymphocytes; (2) Classification based on TCR and BCR chain types: Dual‐TCR αβ T cells, Dual‐TCR γδ T cells, Dual‐BCR B cells, TCR^+^BCR^+^ lymphocytes, γδ^+^αβ^+^ lymphocytes; (3) Classification based on the composition of paired polypeptide chains in the TCR: TCR α^+^α^+^β^+^ T cells (TCR‐ααβ T cells), TCR α^+^β^+^β^+^ T cells (TCR‐αββ T cells), TCR α^+^α^+^β^+^β^+^ T cells (TCR‐ααββ T cells), etc.; (4) Classification based on the composition of paired polypeptide chains in the BCR: BCR H^+^κ^+^κ^+^ B cells (BCR‐Hκκ B cells), BCR H^+^λ^+^λ^+^ B cells (BCR‐Hλλ B cells), BCR H^+^κ^+^λ^+^ B cells (BCR‐Hκλ B cells), BCR H^+^H^+^κ^+^ B cells (BCR‐HHκ B cells), BCR H^+^H^+^λ^+^ B cells (BCR‐HHλ B cells), etc.; (5) Classification based on the classical subsets of the source T or B cells: dual‐TCR Treg T cells, dual‐TCR CD8^+^ T cells, dual‐BCR naïve B cells, etc.

## 2. Proportion and Characteristics of Dual‐BCR B Cells

### 2.1. Dual‐BCR B Cells in Humans and Mice, Transgenic Mice, and Model Mice Under Physiological Conditions (Table S1-1)

The clonal selection theory was proposed in 1959 [1]. Shortly thereafter, Mäkelä and Nossal [9] first reported that clonotypic B cells could simultaneously produce anti‐H and anti‐O antibodies. At that time, the V, D, and J genes of the BCR had not yet been discovered. With the elucidation of the V(D)J rearrangement mechanism and the development of techniques for the preparation and application of κ‐ and λ‐chain antibodies, researchers confirmed the existence of BCR‐Hκλ type B cells at the protein expression level [20]. Progress in studying dual‐BCR B cells in humans and mice has benefited from the establishment of transgenic mouse models and clonal B cell lines, as well as advancements in V(D)J gene detection and sequencing technologies at the mRNA and DNA levels. However, due to differences in technical methods and model selection among laboratories, there is significant variation in the reported proportions of dual‐BCR B cells, as detailed below: (1) In wild‐type mice and various transgenic mouse models [21–30], the proportion of BCR‐HHκorλ type B cells ranges from 0.01% to 4%; the proportion of BCR‐Hκκ (or λλ, κλ) type B cells is ~0.1% to 20%. The proportion of dual‐BCR B cells also varies among different tissues. For example, in 2–6‐month‐old C57BL/6 mice, the proportion of BCR‐Hκλ type B cells was 0.12 ± 0.06% in bone marrow, 0.96 ± 0.29% in spleen, and 1.66 ± 0.82% in the peritoneum, with the highest proportion found in the peritoneum, suggesting a possible role in autoimmune escape mechanisms [24]. (2) For healthy volunteers, the reported proportion of dual‐BCR B cells in peripheral blood ranges from 0.2% to 29% [15, 31–34]. Among these, the incidence of dual heavy chains in a single B cell is significantly lower than that of dual light chains. For instance, Shi et al. [15], using single‐cell BCR sequencing (scBCR‐seq), found that the proportion of single B cells carrying two VH‐DH‐JH rearrangements was 5.90%–8.71%, two VK‐JK rearrangements was 9.71%–13.07%, and two Vλ–Jλ rearrangements was 12.69%–20.07%. Currently, there is no consensus on the proportion of dual‐BCR B cells. Our research team, using scBCR‐seq detection, found that the proportion of dual‐BCR B cells in mice (17%) is higher than in humans (10%) [18], suggesting potential differences in their origin mechanisms and involvement in immune responses. This provides a new research direction for in‐depth exploration of differences in B cell responses among different mammals.

### 2.2. Dual‐BCR B Cells and B Cell‐Related Tumors (Table S1-2)

Given the heterogeneous characteristics of B‐cell‐related lymphomas and plasmacytomas, several research teams have conducted systematic analyses from the perspective of BCR features. As early as 1985, Feddersen and Van Ness [35] discovered that PC3609 plasmacytoma cells exhibited dual immunoglobulin (Ig) κ‐chain rearrangement, with one being a functional rearrangement (κ^+^) and the other non‐functional (κ^−^); whereas PC7043 plasmacytoma cells showed a single Ig κ‐chain rearrangement pattern (Figure S2). Currently, dual‐BCR B cells have been confirmed in various B‐cell‐related tumors. Specifically: In the field of leukemia: B‐cell chronic lymphocytic leukemia patients exhibit simultaneous κ‐ and λ‐chain rearrangements [36]; patients with indolent leukemia harbor three types of B cells: double‐positive (κ^+^λ^+^, 23%), κ single‐positive (κ^+^λ^−^, 24%), and λ single‐positive (κ^−^λ^+^, 20%) [37]. In the field of plasma cell tumors: Plasmacytoma SIPC3282 can simultaneously secrete Ig κ and Ig λ [38]; up to 2% of plasma cell tumor cases exhibit two different heavy or light chain rearrangement patterns [39]; another study showed that 96% of tumor cells in a case of solitary plasmacytoma displayed a κ^+^λ^+^ phenotype [40]; and a κ^+^λ^+^ phenotype was also found in tumor cells from a patient with plasma cell myeloma [41]. In the field of lymphoma: A cohort study of 398 lymphoma patients revealed that 13% of B‐cell lymphoma patients exhibited a dual‐genotype characteristic [42]; studies on some lymphoma cell lines indicated that the proportion of κ^+^λ^+^ phenotype could reach 90% [43]; and in circulating lymphoma cells from a patient with splenic marginal zone lymphoma, 99% of cells showed a κ^+^λ^+^ phenotype [44]. Although the proportional characteristics and underlying molecular mechanisms of dual‐light‐chain BCRs in B‐cell‐related tumors can provide a theoretical basis for the precise diagnosis and targeted therapy of leukemia or lymphoma with unique immune phenotypes [45], it remains unclear whether V(D)J rearrangement‐induced dual‐BCR expression is one of the potential mechanisms triggering B‐cell tumorigenesis. Furthermore, research on the biological effects and regulatory mechanisms of functional versus nonfunctional BCR rearrangements is even scarcer. Apart from B‐cell‐related tumors, Zirakzadeh et al. [46] found that λ^+^κ^+^ memory B cells exist in the tumor tissues of bladder cancer patients, and these cells may exert anti‐tumor effects. This suggests that the anti‐tumor role of dual‐BCR B cells has been underappreciated, positioning them as a promising new direction in B‐cell anti‐tumor research.

### 2.3. Dual‐BCR Cells and Autoimmune Diseases (Table S1-2)

Although the association between dual‐BCR cells and autoimmune diseases was proposed relatively early, related research has primarily focused on systemic lupus erythematosus (SLE) patients and disease model mice. In SLE model mice: B cells expressing dual light chains or dual heavy chains can be detected in the spleen tissues of healthy C57BL/6 mice and MRL/lpr mice, with BCR‐Hκκ B cells accounting for 50% of the total plasma cells and memory B cells in MRL/lpr mice [47]; in hCκ/mCκ (B6 × BALB/c) F1 model mice, ~4% of B cells simultaneously express human and murine κ chains, while in D42 transgenic NZB/NZW mice, the proportion of dual‐light‐chain B cells can reach 13%–40% [48]; compared to healthy mice, the proportion of dual‐BCR B cells is significantly increased in autoimmune disease model mice (Ig κm/h transgenic mice), with BCR‐Hκκ B cells accounting for about 3% [49]. In SLE patients: Using FCM and confocal laser scanning microscopy, Fraser et al. [32] detected BCR‐Hκλ B cells in ~54% of SLE patients; Peterson et al. [16] found that compared to healthy individuals, one‐quarter of SLE patients had a significantly elevated frequency of BCR‐Hκκ B cells, which were mostly B cells expressing VH4‐34 auto‐ antibodies. This frequency strongly correlated with high frequencies of activated naïve B cells and age‐associated B cells. The study also confirmed that BCR‐Hκκ B cells in MRL/lpr mice exhibited enhanced responsiveness to Toll‐like receptors and could simultaneously produce auto‐reactive and non‐auto‐reactive antibodies [16]. To date, whether dual‐BCR B cells represent one of the mechanisms underlying the onset and progression of autoimmune diseases remains to be further clarified.

## 3. Proportion and Characteristics of Dual‐TCR T Cells

### 3.1. Dual‐TCR T Cells in Humans and Mice, Transgenic Mice, and Model Mice Under Physiological Conditions (Table S2-1)

Similar to dual‐BCR B cells, dual‐TCR T cells were initially discovered in clonal T cells. Malissen et al. [10] used Southern blot hybridization, Northern blot hybridization, and Sanger sequencing to confirm the presence of dual functional Vα gene rearrangements and transcription in monoclonal T cells from the spleen of B10. A mice (Figure S3). The two α chains could pair with the β chain, respectively, but only one α chain could recognize both allogeneic major histocompatibility complex (MHC) and self‐MHC‐restricted antigens [10]. Subsequently, researchers verified the existence of dual‐TCR T cells in various transgenic mice, model mice, and normal mice. However, related studies mainly focused on dual TCR Vα chains. The proportions of dual‐TCR T cells reported by different laboratories vary significantly (ranging from 0.01% to 30%), but are generally higher than those of dual‐BCR B cells [50–53]. While analyzing proportions, multiple research teams have explored their origin mechanisms and functions [54–62]. For example, Couez et al. [54] found that CD8^+^ T cell clones from CBA/J mice could carry two functional TCR α genes, but only one paired TCR αβ could be detected on the cell surface; Hardardottir et al. [58] showed that in T cells from transgenic mice expressing two TCRs, most cells had only one TCR capable of specifically recognizing peptides presented by self‐MHC molecules. Extensive studies have confirmed that TCR α chains do not follow the “allelic exclusion” theory during thymic development and rearrangement. For instance, Furutani et al. [63] found that both α chains had in‐frame rearrangements, but ultimately only one α chain polypeptide was expressed on the cell surface; Borgulya et al. [55] found that thymic T cells in αβ TCR transgenic mice attempted to pair with thymic MHC ligands via different TCR αβ combinations, and α chain rearrangement only ceased after positive selection. These studies suggest that allelic exclusion for TCR α chains may occur at the post‐transcriptional level, but its specific mechanism remains unclear.

Unlike TCR α chain rearrangement, TCR β chain rearrangement was once thought to follow allelic exclusion rules. However, van Meerwijk et al. [64] found a considerable proportion of T cells simultaneously expressing two different TCR β chains in the lymph nodes and thymus of TCR Vβ2 and TCR Vβ8.2 transgenic mice, indicating that allelic exclusion is not absolute. Subsequent studies by multiple laboratories investigated the proportion of dual‐TCR β chain T cells in healthy mice [14, 65–67];, with reported proportions around 10%, generally lower than those of dual‐TCR α chain T cells. In transgenic and model mice, the proportion of lymphocytes expressing dual TCR β chains ranged from 1% to 10% [65, 68], while the proportion expressing dual TCR α chains was ~10% to 30% [69, 70]. Our research team observed that the proportion of dual‐TCR Treg cells in different mouse tissues was about 10% and varied by tissue site, suggesting a potential association with the diverse tissue regulatory functions of Treg cells [71].

In studies involving healthy volunteers, the reported proportion of dual‐TCR β chain T cells is ~1% to 10% [18, 72, 73], while the proportion of dual‐TCR α chain T cells may be as high as one‐third [74]. In T cell subset studies, Tuovinen et al. [75] estimated that over 50% of CD4^+^CD25^+^ T cells in healthy adult peripheral blood might express two different TCR Vα chains, and these cells exhibited higher FOXP3 expression levels compared to single‐TCR T cells. Recently, Chen et al. [76] developed a tool (DeRR) specifically designed to identify dual‐TCR T cells in scTCR‐seq and scRNA‐seq data. Analysis of a large sample size (147 samples) of single T cells (over 600,000 cells) revealed that about 17% of cells carried dual TCR α chains and 12% exhibited dual TCR β chains [76].

### 3.2. Dual‐TCR T Cells and Tumors (Table S2-2)

Based on TCR polypeptide chain composition, dual‐TCR T cells can be classified into dual‐TCR αβ T cells and dual‐TCR γδ T cells. Currently, there are relatively few studies directly linking dual‐TCR T cells to tumors themselves. Relevant reports include: clonal T cells from B10. A bone marrow chimeric mice showed rearrangements of two Vβ1 gene segments (14.0 kb and 7.2 kb) [12]; chronic lymphocytosis patients harbored dual‐TCR T cells expressing Vγ4/Vγ5/Vδ1 [77]; γδ T cell lymphoma contained dual‐TCR T cells expressing Vγ3/Vγ9/Vδ1 [78].; and large granular lymphocyte leukemia patients had dual‐TCR T cells expressing Vβ12/Vβ22 and Vα1.4/Vα4.1 [79]. Notably, if malignant T cells simultaneously express two different TCRs on their surface, this may impact the efficacy of therapeutic immunotherapies targeting TCRs [80]. Theoretically, the occurrence of neoplastic dual‐TCR T cells, neoplastic dual‐BCR B cells, and TCR^+^BCR^+^ lymphomas is primarily associated with chromosomal abnormalities, such as translocations, which can lead to disordered V(D)J rearrangements and multiple receptor molecule expression. However, the specific mechanisms require further in‐depth investigation.

Significant progress has been made in studying the anti‐tumor effects of dual‐TCR T cells: Gladow et al. [81] engineered dual‐TCR T cells targeting the melanoma ova257 epitope and gp33 epitope and found they could effectively inhibit the growth of B16‐ova and B16‐gp33 melanoma cells; Weinhold et al. [82] constructed dual‐TCR CD8^+^ T cells targeting a tumor‐specific antigen and a pancreatic self‐antigen, demonstrating that one TCR specifically recognized the tumor antigen while the other recognized the pancreatic self‐antigen, exerting anti‐tumor effects without causing severe side effects; Jang et al. [83], using a mouse model carrying a TCR α reporter gene for studying 6727 sarcoma and B16F10 melanoma, found a significantly increased proportion of dual‐TCR T cells among tumor‐infiltrating lymphocytes (TILs), and these cells showed a preferential response against B16F10 melanoma.

The advent of combined scRNA+TCR‐seq technology provides new technical support for analyzing the anti‐tumor mechanisms of dual‐TCR T cells [84]. Examples of related research include: Yao et al. [85] detected clonal expansion of dual‐TCR T cells in the tumor microenvironment (TME) of nasopharyngeal carcinoma patients; our team [86] discovered migratory dual‐TCR T cells in the peripheral blood, tumor tissue, and adjacent normal tissue of non‐small cell lung cancer patients. However, Gao et al. [17] found T cells expressing two or three TCR α chains and TCR β chains in patients with T‐cell large granular lymphocytic leukemia, but it remains unclear whether these cells originate from neoplastic T cells or anti‐tumor T cells.

Currently, using scRNA+TCR‐seq technology combined with tools like MixTCRpred enables precise screening and systematic analysis of dual‐TCR T cells in patients [87]; artificially engineered dual‐specific TCR tool cells have shown potential application value in treating diseases like refractory leukemia [88]. Furthermore, Chen et al. [76], through integrated analysis of public scTCR‐seq and scRNA‐seq databases, found that the frequency of dual‐TCR T cells in cancer patients was significantly higher compared to other diseases. These studies collectively suggest that dual‐TCR T cells may play important roles in tumor response or regulation.

### 3.3. Dual‐TCR T Cells and Autoimmune Diseases (Table S2-2)

Dual‐TCR T cells have long been considered a cell population that escapes tolerance selection during T cell development and is exported to the periphery. They may be closely associated with the onset and progression of autoimmune diseases. For example, Furutani et al. [63] identified autoreactive Vα4‐Jα11.2 and Vα5‐JαTA13 helper T cell clones in C3H mice; Padovan et al. [74], using anti‐human Vα monoclonal antibodies, detected that up to one‐third of mature T cells expressed dual Vα chains, and such cells possessed the capacity to participate in heterologous and autoimmune reactions. In transgenic mouse studies, reports linking dual‐TCR T cells to autoimmune diseases are relatively abundant [89–91]. For instance, Ji et al. [90] demonstrated in myelin basic protein (MBP)‐specific TCR α8 Vβ8.8 transgenic mice that dual TCRs could recognize both MBP and viral antigens.

The association between dual‐TCR T cells and diabetes has garnered widespread attention. For example, Elliott and Altmann [92] found that dual‐Vα T cells might play a significant role in the development of autoimmune diseases like non‐obese diabetes; Fossati et al. [93] showed that activation of the second TCR on the surface of diabetogenic T cells could trigger diabetes onset. Regarding the mechanism, Schuldt et al. [94] found that dual‐TCR T cells could restrict the differentiation of autoreactive thymocytes into regulatory T cell (Treg) lineage, ultimately resulting in a cell phenotype with lower tolerance levels and a higher propensity to induce autoimmune reactions.

Recent studies indicate that in ankylosing spondylitis (AS) patients, dual‐TCR‐expressing memory T cells, pathogenic helper T cells 17 (pTh17), and Treg subsets may participate in the pathogenesis of AS [95]; in studies of tertiary lymphoid structures (TLS) in pemphigus, a certain proportion of dual‐receptor CXCL13^+^CD4^+^ T cells were found [96]; Kawasaki disease (KD) is closely related to infection and autoimmune reactions, and our research found that dual‐TCR T cells are involved in the pathogenesis of KD and are associated with the therapeutic response to intravenous immunoglobulin (IVIG) [97]. Additionally, Chen et al. [76], analyzing shared large‐sample single‐cell sequencing data, also found that the distribution of dual‐TCR T cells in autoimmune disease patients positively correlated with disease duration.

Theoretically, T cells expressing dual TCRs are more prone to escape self‐tolerance regulation mechanisms, posing a potential risk to immune homeostasis. This characteristic provides important theoretical support for exploring new mechanisms underlying the development of autoimmune diseases.

### 3.4. Dual‐TCR T Cells and Infectious Diseases or Transplant Rejection (Table S2-2)

Although dual‐TCR T cells generated from V(D)J allelic exclusion escape may play detrimental roles in lymphoma and autoimmune diseases, they could have positive effects in individual anti‐infection responses. This is because dual‐TCR T cells can expand the diversity of the TCR repertoire [98]. Current reports on the association between dual‐TCR T cells and infectious diseases mainly include: ~10% of CD8^+^ T cells in the lungs of influenza virus‐infected mice express two highly transcribed TCR α chain mRNAs [99]; after isolating antigen‐specific T cells from the peripheral blood of subjects who cleared hepatitis C virus infection, it was found that among 89% of TCR αβ^+^ T cells, 18% expressed dual α chains and 7% expressed dual β chains [14]; dual‐TCR T cells can suppress eosinophil responses via IFN‐γ and, through microbiota‐induced Th17 cell differentiation, promote neutrophil inflammatory responses, leading to biased OVA‐specific immune responses in DO11.10 mice [62].

Theoretically, enhanced responsiveness of dual‐TCR T cells to self‐antigens might simultaneously increase their responsiveness to alloantigens. Padovan et al. [74] first proposed the possibility of dual‐TCR T cells participating in alloreactivity; Simpson et al. [100] first detected dual‐TCR T cells capable of mediating allogeneic skin graft rejection in TCR transgenic mice specific for the influenza virus NP366‐374 epitope/H‐2Db molecule. It has now been confirmed in graft‐versus‐host disease (GVHD) model mice [101], symptomatic GVHD patients [102], and hematopoietic stem cell transplant patients with GVHD [103] that the proportion of dual‐TCR T cells is significantly increased and they participate in transplant immune response effects.

Regarding mechanistic studies, Zhou et al. [104] found in SCID‐bg mice that most dual‐TCR T cells were distributed among activated T cells, memory T cells, and regulatory T cell (Treg)subsets; Daniel et al. [105] confirmed the existence of a T cell subset expressing dual TCR α chains (Vα2 and Vα4) and a single TCR β chain (Vβ1) among naturally occurring alloreactive T cells. Both in vitro and in vivo experiments have confirmed that alloantigen stimulation can preferentially activate and expand dual‐TCR T cells [106]. This suggests that investigating the pathogenesis of acute GVHD by targeting the elimination of dual‐TCR T cells opens a new direction for research in the field of transplant immune responses [107].

## 4. Other Dual‐Receptor Lymphocytes

### 4.1. Dual‐TCR γδ T Cells

Aside from the dual‐γ‐chain T cells found in lymphomas mentioned earlier [77, 78], research on dual‐TCR γδ T cells actually predates that on dual‐TCR αβ T cells. However, the dual γ‐chain expression observed in early studies of mouse clonal T cells may not have originated from inclusive rearrangement of γ‐chain V‐J alleles on both chromosomes [108–111]. The reason lies in the structure of the mouse TCR γ‐chain locus, which contains four independent V, J, C gene clusters arranged in both forward and reverse orientations. Consequently, a single chromosome can undergo both forward “looping‐out rearrangement” and “inversion rearrangement,” retaining two (or more) different types of VJC rearrangements on the chromosome that can be expressed through transcription [112]. For example, Heilig and Tonegawa[108], using Southern blot hybridization, confirmed the presence of functional rearrangements for γ‐chain V1‐J4‐C4 and V2‐J2‐C2 in clonal T cells. Davodeau et al. [113] were the first to confirm at the protein level the presence of T cells in human peripheral blood simultaneously expressing γ‐chain V9 and V4, which could pair with δ‐chain subfamilies V1, V2, V3, and V5, respectively. Peyrat et al. [114] first reported a high proportion of T lymphocytes expressing δ‐chain V1 and V3 in the peripheral blood of healthy humans. Compared to dual‐TCR αβ T cells, dual‐TCR γδ T cells show no significant association with autoreactivity [115]. In the field of anti‐infection immune responses, Orsini et al. [116] found that dual‐TCR T cells expressing γ‐chain V4^+^V9^+^ and δ‐chain V1^+^ might be involved in the response to *Staphylococcus aureus* superantigen; Taupin et al. [117] also found that T cells expressing dual δ‐chains in the peripheral blood of individuals infected with human immunodeficiency virus (HIV) might participate in the immune response. Given that the function of γδ T cells has been shown to lie between innate and adaptive immune responses [118], research on dual‐TCR γδ T cells will provide new insights and directions for elucidating novel regulatory mechanisms linking innate and adaptive immunity.

### 4.2. γδ+αβ+ Lymphocytes

The primary decision faced by lymphoid stem cells migrating to the thymus is whether to initiate γδ T cell rearrangement or αβ T cell rearrangement, and the underlying regulatory mechanisms for this process are not fully understood. Early studies identified a small number of T cells in the mouse thymus capable of undergoing both γδ and αβ rearrangements: Ishida et al. [119] detected αβ TCR gene rearrangement in TCR γ transgenic mice; Livak et al. [120] confirmed a high proportion of δ‐chain VDJ gene rearrangement in mouse thymic αβ T cells; and Bowen et al. [121] found that the Vγ2^+^Cβ^+^ T cell subset in mice participates in the differentiation and development of αβ T cells. Hochstenbach and Brenner [122] first discovered the paired expression of δ‐chain and β‐chain dimers in a T‐cell leukemia cell line (DND41). Subsequent studies confirmed that simultaneous rearrangement of αβ T cells and γδ T cells also occurs in human peripheral blood [114] and thymic tissue [123]: Bosco et al. [124] found a high frequency of TCR β rearrangement in γδ T cells, and this rearrangement process could be involved in regulating αβ T cell development; Pellicci et al. [125] demonstrated that human Vδ1^+^αβ^+^ T cells can recognize CD1d molecules and peptide‐ and lipid‐based antigens. Recently, Edwards et al. [126] identified Vγ4^+^TCRδ^+^TCRβ^+^ T cells in the thymus of 16‐day‐old mice. These cells possess the ability to recognize MHC‐restricted peptide antigens (a hallmark of αβ T cells) and also respond to inflammatory stimuli, exerting pro‐inflammatory effects (a hallmark of γδ T cells), playing important roles in disease models such as *S. aureus* infection and experimental autoimmune encephalomyelitis [126]. These findings provide a new research direction for deciphering the regulatory mechanisms governing the choice between γδ and αβ T cell rearrangement in thymic lymphoid stem cells.

### 4.3. TCR^+^BCR^+^ Lymphocytes

The germline V(D)J gene rearrangement of TCR and BCR follows the same regulatory molecular mechanisms and rearrangement rules. However, under physiological conditions, B cells in the bone marrow undergo only BCR V(D)J rearrangement and do not simultaneously initiate TCR V(D)J rearrangement; conversely, T cells in the thymus undergo only TCR V(D)J rearrangement. The complex regulatory mechanisms behind this strict rearrangement pattern are currently unclear. The special phenomenon of simultaneous TCR and BCR rearrangement within a single cell was first discovered by Hanawa et al. [127] in 1995 in a case of splenic lymphoma, namely TCR^+^BCR^+^ lymphocytes (Figure S4). In the field of lymphoproliferative disorders and lymphomas, related reports include: cells possessing both T‐cell and B‐cell genotypes found in pyothorax‐associated lymphoma [128]; detection of TCR gene and immunoglobulin heavy chain gene rearrangement in a patient with angioimmunoblastic T‐cell lymphoma [129]; confirmation of cells simultaneously expressing BCR and TCR in canine germinal center blast lymphomas [130]; discovery of dual rearrangement of immunoglobulin κ‐chain gene and TCR genes in large B‐cell lymphoma [131]; and detection of simultaneous TCR and immunoglobulin heavy chain (IgH) rearrangement in primary cutaneous proliferative disorders [132]. These findings suggest that the pathogenesis, clinical diagnosis, and treatment strategies for lymphoproliferative disorders and lymphomas may be closely related to the aberrant initiation of V(D)J rearrangement. Besides lymphomas, Ahmed et al. [133] used FCM to detect ~2.2% TCR^+^BCR^+^ lymphocytes in the peripheral blood of three patients with type 1 diabetes (T1D) and about 0.4% in healthy controls. Further research indicated that TCR^+^BCR^+^ lymphocytes in the peripheral blood of T1D patients were enriched with a shared BCR clonotype capable of encoding potent autoantigens, thereby stimulating the activation of autologous CD4^+^ T cells [134]. This result suggests that TCR^+^BCR^+^ lymphocytes also exist under physiological conditions, and their increased proportion may be associated with the onset and progression of autoimmune diseases, presenting a new challenge for research on V(D)J rearrangement mechanisms and their biological effects.

## 5. Mechanisms of Dual‐Receptor Lymphocyte Generation

### 5.1. V(D)J Allelic Exclusion or Inclusion Rearrangement Models and Regulatory Molecules

The discovery and annotation of V(D)J genes, along with the “12/23 rearrangement rule” governing recombination signal sequences (RSS), have profoundly revealed the genetic and molecular basis for the high specificity and diversity of adaptive immune responses. The rearrangement patterns of germline V(D)J genes and the mechanisms of tolerance selection determine whether a lymphocyte ultimately expresses a single receptor or dual receptors. However, despite over 50 years of research, the core mechanisms and key regulatory factors controlling allelic exclusion or inclusive rearrangement remain incompletely elucidated.

Alt et al. [135] first confirmed the functional rearrangement and exclusion phenomenon of immunoglobulin (Ig) κ and λ chains in mice, and further clarified in 1984 that D‐J rearrangement precedes V‐DJ rearrangement in B cells. Their studies also found that the formation of the first functional V‐DJ rearrangement could block other V gene segments from participating in DJ rearrangement, laying an important foundation for research on feedback regulation of V(D)J rearrangement [135, 136]. In 1985, Weaver et al. [137] demonstrated that introducing a functional Ig μ chain gene into the mouse genome could significantly inhibit the rearrangement of endogenous genes in B cells, thereby initiating systematic exploration of the molecular regulatory mechanisms of V(D)J allelic exclusion rearrangement. Various key molecules regulating V(D)J allelic exclusion rearrangement have since been identified: the formation of the pre‐TCR complex can activate protein kinase C (PKC), which in turn mediates allelic exclusion at the TCR β locus [138]; in CD4^+^CD8^+^ double‐positive thymic T cells, cis‐acting elements can regulate the accessibility of Vβ gene segments, to some extent ensuring the specific occurrence of allelic exclusion rearrangement [139]; Ets‐1 ensures that forced activation of pre‐TCR signals does not block the rearrangement of endogenous TCRβ genes, preventing an abnormal increase in the proportion of thymocytes expressing two TCR β chains [140]; recombination‐activating gene 1 (RAG‐1) and ataxia telangiectasia mutated protein (ATM) can synergistically regulate monoallelic rearrangement and nuclear positioning of immunoglobulin loci [141], and ATM can prevent inter‐allelic cross‐rearrangement, maintaining genomic stability [142].

Fondell et al. [143] first observed allelic inclusion rearrangement at the Vα‐Jα locus in TCR, proposing that cells whose germline genes had already undergone functional Vα‐Jα rearrangement could still undergo functional or non‐functional “secondary rearrangements.” Their study found that one TCR α allele in a clonal cell line even underwent three rearrangements, suggesting that T cells could bypass allelic exclusion rearrangement at the TCR α locus during early development [143]. Subsequently, Elliott [52] showed that if the Vα chain expressed by a T cell was inefficient in positive selection, the probability of it co‐expressing a second Vα chain increased significantly. This disorder in TCR Vα chain pairing selection might be a significant trigger for allelic inclusion rearrangement.

Studies on regulatory molecules for allelic inclusion rearrangement of the TCR β chain found that TCR β chain rearrangement begins on one allele; rearrangement of the other allele only initiates if the first fails. In pre‐TCRα chain (pre‐TCRα, pTα) gene knockout (pTα^−^/^−^) mice, a high proportion of thymocytes expressing two functional TCR β alleles were detected, suggesting that under physiological conditions, the pTα chain plays a key regulatory role in allelic exclusion rearrangement at the TCR β locus [69]. Additionally, the quality of TCR Vβ gene RSS (measured by recombination signal information content score) is closely related to the efficiency of allelic exclusion rearrangement: if the Vβ‐RSS quality is poor, other functional TCR β alleles can participate in rearrangement assembly and expression before the TCRβ protein generates feedback inhibitory signals [144, 145].

To explain the complex regulatory process of V(D)J allelic exclusion rearrangement, scholars have proposed various models [6], mainly including the asynchronous rearrangement model, feedback inhibition model, and stochastic model. The complexity of asynchronous rearrangement in V(D)J allelic exclusion rearrangement may be related to chromatin structure accessibility and histone modifications; DNA demethylation and epigenetic modifications can regulate the initial rearrangement of a single allele and block secondary gene rearrangement, while mechanisms like feedback signal transduction from alleles, locus contraction and expansion, and heterochromatin recruitment are closely related to multiple regulatory molecular pathways [6–8]. The feedback inhibition model relies on feedback signals from the rearrangement of “intermediate products” to inhibit further rearrangement of the second allele [146]. This feedback mechanism can block V‐to‐DJ rearrangement but does not affect D‐to‐J rearrangement [147]. Observations of allelic inclusion rearrangement in different transgenic mouse models by multiple laboratories suggest an “incomplete feedback mode": inserting two independent IgH alleles into transgenic mice revealed that in‐frame V–D–J rearranged gene segments could successfully rearrange more than one functional allele [148]; after introducing a third allele, all three alleles could undergo rearrangement [149]; in dual‐TCR transgenic mice, the regulatory mechanism for functional allelic exclusion rearrangement might operate through controlling TCR assembly rather than relying on feedback at the transcriptional or expression level [150].

Currently, new rearrangement hypotheses and mechanisms are still being continuously explored and supplemented. For instance, Farcot et al. [151] proposed a model based on dynamic continuous time to simulate the D‐J and V‐DJ rearrangement and feedback inhibition processess; Outters et al. [5] systematically summarized stochastic and probability models used to explain allelic exclusion rearrangement.

At present, the regulatory molecules and mechanisms of V(D)J allelic exclusion or inclusion rearrangement still cannot fully explain why allelic exclusion rearrangement mainly occurs for the TCR β chain and BCR heavy chain; the specific molecules and key conditions initiating this process also remain unclear. Understanding the patterns of allelic exclusion and inclusion rearrangement is crucial for elucidating the origin of single and dual‐receptor lymphocytes, yet this remains a core bottleneck in current research on the proportion, function, and generation mechanisms of dual‐receptor lymphocytes [152].

The origin mechanisms of dual‐receptor lymphocytes are extremely complex. Current understanding of V(D)J allelic exclusion/inclusion rearrangement on homologous chromosomes remains preliminary. In human and mouse T and B cells, the seven polypeptide chains are distributed across six chromosomes. The TCR α chain (TRA) and TCR δ chain (TRD) gene loci are located on the same chromosome. After V(D)J gene rearrangement, although experimental evidence suggests they might pair with each other (e.g., in TCR^+^BCR^+^ lymphocytes or γδ^+^αβ^+^ lymphocytes), research on their origin mechanisms is almost entirely blank.

### 5.2. Central and Peripheral Self‐Tolerance Selection and Dual‐Receptor Lymphocytes

Although germline V(D)J allelic exclusion rearrangement escape (i.e., allelic inclusion rearrangement) is one of the primary mechanisms leading to the generation of dual‐receptor lymphocytes, it is now established that the rearrangement form for TCR α chains and BCR light chains is allelic inclusion rearrangement. The core feature of “one lymphocyte, one type of receptor” is primarily maintained through stringent central self‐tolerance selection. That is, only lymphocytes with one successfully paired TCR α and β chain (or one BCR light and heavy chain) pass selection (avoid apoptosis) and are exported to the periphery. The detailed mechanisms of this stringent selection are currently unclear, and even less is known about the mechanisms of selection failure (i.e., where two TCR α chains or two BCR light chains participate in pairing).

The association between receptor editing in central and peripheral compartments and dual‐receptor lymphocytes has garnered widespread attention. Receptor editing occurs primarily in B cells and is an important pathway for shaping the B cell antibody repertoire, as well as a core mechanism for B cells to maintain tolerance to self‐antigens. Among these, the editing and replacement of κ chains is one of the important sources of dual‐BCR B cells [27]. Studies in the LamH‐C μ‐Ig‐H/Vκ8Jκ5L transgenic mouse model, focusing on receptor editing via nontargeted κ chain alleles, suggest that autoreactive dual‐BCR B cells may originate from laminin‐triggered RAG activity and light chain editing [23]. Research in the 3‐83Igi H‐2d/B1‐8/3‐83Igi H‐2b transgenic mouse model found that receptor editing, while generating non‐self‐reactive antigen receptors (Ag receptors), does not inactivate the self‐antibody‐encoding gene in every autoreactive B cell. Instead, it can induce the simultaneous expression of the original autoreactive and novel non‐self‐reactive Ag receptors, hiding the self‐antibody intracellularly and presenting a surface‐tolerant phenotype [26].

In studies of normal mice, Casellas et al. [28] found that 10% of mature B cells co‐expressed two Ig κ chain alleles, a mechanism derived from receptor editing. Rice et al. [153] confirmed that receptor editing in B cells is not confined to immature B cells in the bone marrow. Mature B cells in the mouse periphery (spleen) can re‐initiate RAG gene expression upon activation by self‐reactive antigens, correcting or eliminating autoreactive B cells through light chain receptor editing [153], suggesting that peripherally induced tolerant B cells may also be a source of dual‐BCR B cells.

Typically, T cells do not alter their tolerance state via receptor editing mechanisms. However, it is noteworthy that inducing tolerance to one TCR in a dual‐TCR T cell does not affect the response of the other TCR [154] This finding provides experimental evidence for the self‐immune tolerance escape of dual‐TCR T cells and their simultaneous response to antigens.

### 5.3. Exploration of New Mechanisms for Dual‐Receptor Lymphocyte Origin via scRNA+V(D)J‐seq

The integrated application of single‐cell RNA sequencing combined with V(D)J sequencing (scRNA+V(D)J‐seq) technology has opened new perspectives for studying the proportion and function of dual‐receptor lymphocytes and provides a unique opportunity to deeply investigate the mechanisms of allelic exclusion and allelic inclusion rearrangement. This technology allows for the simultaneous monitoring, at high‐throughput single B or T cell resolution, of the proportion and compositional characteristics of dual receptors, along with their corresponding subset distribution and transcriptomic expression patterns. Currently, this technology is widely used to study dual‐receptor lymphocytes in peripheral and central immune organs of mice and humans under physiological and pathological conditions. Data sharing among laboratories has further propelled the development of this emerging field [13–19, 155–159].

Our high‐throughput single‐cell studies found that dual‐BCR B cells and dual‐TCR T cells occupy stable proportions under physiological conditions, and the proportion of dual‐receptor lymphocytes is higher in mice than in humans [18, 19]. It is crucial to emphasize that the TCR or BCR mRNA transcript sequences captured by scVDJ‐seq exist in single‐chain, dual‐chain, and multi‐chain forms. Their proportions, origins, underlying mechanisms, and biological significance currently lack systematic study. Moreover, analysis of dual‐receptor lymphocytes based on mRNA levels still requires further validation at the DNA level (e.g., using scATAC+VDJ‐seq technology) and protein expression level. Our single‐cell sequencing results for human and mouse TCR and BCR show that “a single lymphocyte contains mRNA transcripts for three (or more) V(D)J rearrangements within one chain type (TCRα, TCRβ, BCR heavy chain, BCR light chain) (Figure 2).“ This contradicts the classical rule that “only two alleles on the two chromosomes (paternal and maternal) participate in rearrangement and expression in a single T or B cell.” Theoretically, although multiple rounds of allelic co‐inclusion rearrangement can occur on a single chromosome, the second rearrangement removes the first V(D)J sequence via “looping out,” ultimately retaining only two functional V(D)J rearrangements for mRNA transcription [3]. Considering the positions and order of V, J, D genes on human and mouse IGH and TR loci, along with the 12/23 rule, allelic exclusion rule, and the D‐J before V‐DJ rearrangement rule [3, 6, 8, 160, 161] and based on analysis of complementarity‐determining region 3 (CDR3) sequence characteristics from extensive scVDJ‐seq data, we propose a potential new mechanism whereby a single chromosome can undergo two functional rearrangements and transcriptions [18, 19] (Figure S1). That is, the inversely oriented human TRBV30 gene (mouse TRBV31 gene) in the TRB locus might undergo an inverse “inversion rearrangement,” allowing two functional rearrangements to occur on a single chromosome and both be retained on the chromosome for transcription. Inversely oriented V genes present in the IG locus might have a similar mechanism. However, the specific mechanisms and frequencies of V(D)J “looping‐out rearrangement” and “inversion rearrangement” remain unclear. Studies by Zhang et al. [162], Hill et al. [163] found that chromatin ring extrusion plays an important role in IGH locus contraction and physiological V(D)J rearrangement, while “inverse D‐J rearrangement” upstream of the 5^′^ 12RSS is relatively rare, suggesting that the mechanism of secondary functional rearrangement of the IGH chain on a single chromosome requires further in‐depth study. For example, whether the secondary V–D–J (IGH, TRB) or V–J (TRA, IGK, IGL) functional rearrangement on a single chromosome originates from mRNA expression derived from a “functional looping‐out rearrangement circle” is a hypothesis that still needs further verification [18, 19]. When using scRNA+V(D)J‐seq for high‐throughput single‐cell dual‐receptor studies, how to integrate single‐cell transcriptomics with epitope sequencing (scCITE‐seq) and Mass Spectrometry‐based Single Cell Proteomics technology to conduct protein expression and functional studies at the high‐throughput single‐cell level is a research direction urgently needing development.

Due to differences in the number of V, D, J, and C genes within mammalian TR and IG loci and their varying cluster arrangements, the mechanisms and frequencies of V(D)J allelic exclusion or inclusion rearrangement also differ significantly. For example, in the mouse IGL locus, J1‐C1, J2‐C2, J3‐C3, J4‐C4 are arranged as four independent gene clusters along the embryonic DNA strand, with V2 and V3 genes located in one cluster and V1 in another independent cluster. This unique structure allows at least two independent V–J–C rearrangements and transcriptions of V genes to occur on a single mouse IGL chromosome. It is crucial to emphasize that experimental evidence of “specific mRNA expression within a single T or B cell” still requires further verification with experimental results from the DNA rearrangement level and protein expression level of V(D)J.

## 6. Effects of Dual‐Receptor Lymphocytes

Currently, a core limitation of experimental studies supporting the existence of dual‐BCR B cells and dual‐TCR T cells is their predominant focus on V(D)J gene rearrangement and the transcriptional level, while research at the protein expression and functional levels remains insufficient. For studies on dual‐BCR B cells, existing techniques often rely on κ‐chain‐ and λ‐chain‐specific antibodies, limiting analysis to κ^+^λ^+^ type dual‐BCR B cells. For dual‐TCR T cell research, studies primarily depend on specific antibodies targeting different V gene families of the TCR β‐chain and α‐chain, allowing for the screening and identification of a limited number of dual‐TCR T cells through finite antibody pairing combinations.

Progress in research on the functional molecular mechanisms and effects of dual‐receptor lymphocytes is as follows: Within the splenic CD4^+^ T cell population of DO11.10 TCR transgenic mice, antigen‐specific dual‐TCR T cells targeting intestinal antigens have been identified; these cells can promote the development and regeneration of IL‐10/IFN‐γ‐secreting memory/effector cell populations [164]. Intracellular cytokine staining confirmed that dual‐TCR T cell subsets within virus‐specific Th1 or Th2 populations exhibit unique cytokine secretion patterns and functional characteristics [165]. Studies in transgenic mouse models indicate that dual‐TCR T cells, after specific treatment, can induce immune tolerance, i.e., they no longer mount an immune response to antigens that would normally trigger an autoimmune response [154]. T cells simultaneously expressing two different TCRs (specific for ova257 and gp33 antigens, respectively) can be specifically activated by the corresponding antigen peptides and secrete IFN‐γ, both contributing to anti‐tumor immune responses [81]. The functional activity of dual‐TCR T cells is regulated by the intrinsic properties of the TCR complex and is closely related to antigen affinity and the number of specific TCRs expressed on the cell surface [166]. Dual‐TCR T cells show significantly elevated expression of the activation marker CD69 and can secrete more pro‐inflammatory cytokines IFN‐γ and interleukin‐17A (IL‐17A) [102]. Mucosal‐associated invariant T (MAIT) cells, in addition to expressing the canonical TCRα chain (TRAV1‐2), can simultaneously express two non‐canonical TCRα chains; when these MAIT cells bind to specific antigens (5‐OP‐RU), they generate specific immune responses through the synergistic action of canonical and non‐canonical TCRα chains [167].

Experimental evidence demonstrating that dual‐BCR B cells and dual‐TCR T cells can perform dual functions provides important theoretical reference for applied research on bispecific antibodies [168] and bispecific chimeric antigen receptor T cells (CAR‐T) [169]. In recent years, the successful construction of dual‐TCR T cell animal models [170] and their effective application in investigating the anti‐tumor roles and mechanisms of dual‐TCR T cells [83] have provided feasible technical solutions for in‐depth research on dual‐receptor lymphocytes.

Previous studies indicate that the proportion of dual‐receptor lymphocytes is significantly elevated in autoimmune diseases, tumors, infectious diseases, and transplant rejection, with some functions already confirmed, suggesting their potential involvement in the onset and progression of these diseases. However, their specific effector roles and molecular mechanisms require further validation. Concurrently, existing research finds differences in the proportion of dual‐TCR T cells across different tissue sites [171], and the proportion of dual‐receptor lymphocytes shows dynamic changes across different age stages [172], highlighting the complexity and difficulty of studying dual‐receptor lymphocyte effects. Furthermore, while most current transgenic and model mice are constructed based on the TCR β chain, recent studies confirm that allelic inclusion recombination in the dual‐TCRα chain VJ region can lead to a significantly higher number of dual‐TCR ααβ T cells compared to dual‐TCR ββα T cells, suggesting that dual‐TCR ααβ T cells hold greater value for in‐depth study [55, 173, 174]. In recent years, the establishment of dual‐TCRα reporter mouse models and their application in fields like oncology [83, 170], along with research advances such as the “temporal rules” of proximal and distal VJ gene rearrangement within the thymus and the preferential distribution of VJ gene rearrangement products among different T cell subsets [155], have provided new technical support and research directions for studying the effects and mechanisms of dual‐TCR ααβ T cells.

## 7. Conclusion

Dual‐receptor lymphocytes can expand the body’s antigen receptor repertoire, enhancing the dual recognition and capture capabilities of T cells and B cells for specific antigen epitopes. However, they may also allow T cells and B cells to escape self‐tolerance selection, thereby increasing the risk of autoimmune responses. Currently, the molecular mechanisms underlying key biological processes such as “antigen epitope molecular capture” and “immune synapse formation with target cells” in dual‐TCR T cells and dual‐BCR B cells are not fully elucidated. Recent studies report that TCR^+^BCR^+^ lymphocytes may be associated with the development of diabetes [133]. These cells are also expressed in small numbers under physiological conditions, and research suggests they originate from progenitor/pre‐B cells (pro/pre B cells) [175]. However, the molecular mechanisms for synchronous TCR and BCR rearrangement and the co‐expression of CD3 and CD19 remain largely unexplored. Additionally, research on the origin mechanisms, effector functions, and regulatory networks of dual‐TCR MAIT cells, dual‐TCR γδ T cells, γδ^+^αβ^+^ lymphocytes, and more refined subsets (e.g., dual‐TCR Treg cells) is still in its infancy.

Although experimental evidence confirms that dual‐receptor lymphocytes exist not only under physiological conditions but also in model mice and various pathological contexts—including infectious diseases, T/B cell‐related lymphomas, graft rejection, autoimmune diseases, and neoplastic diseases—current understanding of their cellular proportions, origin pathways, effector mechanisms, and regulatory principles remains relatively limited. Key scientific questions urgently needing resolution include: What are the detailed mechanisms and regulatory molecules governing allelic exclusion and allelic inclusion during V(D)J rearrangement, and can the generation of dual‐receptor lymphocytes be artificially regulated? To address these research gaps, it is necessary to deepen the understanding of genetic‐level and chromatin‐level modifications and regulatory mechanisms governing “V(D)J allelic co‐inclusion and allelic exclusion rearrangement for TCR and BCR.” This includes refining the regulatory mechanisms for “functional and non‐functional rearrangements” at the transcriptome level and elucidating the post‐translational pairing and assembly rules for TCR and BCR polypeptide chains. The development of single‐cell sequencing technology has provided technical support for high‐throughput transcriptome analysis of individual lymphocytes in humans, mice, and various model organisms. However, research techniques and comprehensive systems for studying DNA recombination levels and protein expression levels are still relatively lacking.

The natural existence of dual‐receptor lymphocytes under physiological conditions has already provided an important theoretical foundation and novel research ideas for the preparation and clinical application of bispecific antibodies and dual CAR‐T cells [176, 177]. In the future, establishing stable research models for dual‐TCR T cells, dual‐BCR B cells, TCR^+^BCR^+^ lymphocytes, and γδ^+^αβ^+^ lymphocytes, and screening, identifying, and constructing engineered dual‐receptor lymphocytes derived from pathological states will be the most pressing research directions in this field. Looking ahead, in‐depth analysis of dual‐receptor lymphocytes will not only provide an important supplement to the classic clonal selection theory but also open broad prospects for the clinical application of specific immune responses.

**Nomenclature**
BCR: B‐cell receptor
TCR: T‐cell receptor
V: variable gene
D: diversity gene
J: jointing gene
FCM: flow cytometry
PCR: polymerase chain reaction
scRNA+V(D)J‐seq: single‐cell RNA‐sequencing+ V(D)J‐sequencing
GVHD: graft‐versus‐host disease
HSCT: hematopoietic stem cell transplant
IFN‐γ: Interferon‐γ
IL‐17A: Interleukin‐17A
PBL: peripheral blood lymphocyte
RAG: recombination activating gene
RSS: recombination signal sequence
SLE: systemic lupus erythematosus
T1D: Type Ⅰ diabetes
MAIT: mucosal associated invariant T
TCR‐ααβ T: TCR α^+^α^+^β^+^ T cell
TCR‐‐αββ T: TCR α^+^β^+^β^+^ T cell
TCR‐‐ααββ T: TCR α^+^α^+^β^+^β^+^ T cell
BCR‐‐Hκκ B: BCR H^+^κ^+^κ^+^ B cell
BCR‐‐Hλλ B: BCR H^+^λ^+^λ^+^ B
BCR‐‐Hκλ B: BCR H^+^κ^+^λ^+^ B
BCR‐HHκ B: BCR H^+^H^+^κ^+^ B
BCR‐‐HHλ B: BCR H^+^H^+^λ^+^ B.

## Author Contributions

Qi Peng, Lanwei Zhu, and XiaoPing Lu were responsible for literature retrieval, sorting and analysis, and the making of charts and tables; Xinsheng Yao was responsible for the writing of articles.

## Funding

The National Natural Science Foundation of China (82471630) and Guizhou Provincial High‐level Innovative Talents Project [No. (2018) 5637] funded this study.

## Disclosure

All authors read and approved the final manuscript.

## Ethics Statement

The authors have nothing to report.

## Consent

The authors have nothing to report.

## Conflicts of Interest

The authors declare no conflicts of interest.

## Supporting Information

Additional supporting information can be found online in the Supporting Information section.

## Supporting information


**Supporting Information** Figure S1. Based on the composition and arrangement order of VDJC genes in the murine TRB locus, we constructed a schematic diagram detailing the sequential processes of VDJ recombination on a single mouse chromosome under the framework of classical VDJ recombination rules. This includes the inversion recombination involving the inverted V31 gene, the loop‐out recombination involving the forward Vx gene, as well as the canonical sequential order of D‐J rearrangement followed by V‐DJ rearrangement. Furthermore, we propose a potential mechanism by which two rounds of functional VDJ recombination and subsequent transcription can be accomplished on a single chromosome in accordance with classical recombination rules. Figure S2. Example Visualization of the Results from the 1985 Dual BCR Study (Feddersen and Van Ness [35], Proceedings of the National Academy of Sciences, 1985). Figure S3. Example Visualization of the Results from the 1988 Dual TCR Study (Triebel et al. [11], Journal of Immunology, 1988). Figure S4. Example Visualization of the Results from the 1995 TCR+BCR+ Study (Hanawa et al. [127], Leukemia & Lymphoma, 1995). Table S1‐1. In physiological conditions of humans and mice: The main overview and progress of the research objects, methods, and characteristics of dual BCR B cells/TCR + BCR + lymphocyte. Table S1‐2. In pathological conditions of humans and mice (B‐cell associated tumor; autoimmune diseases, etc.): The main overview and progress of the research objects, methods, and characteristics of dual BCR B cells/TCR + BCR + lymphocyte. Table S2‐1. In physiological conditions of humans and mice: The main overview and progress of the research objects, methods, and characteristics of dual TCR T cells. Table S2‐2. In pathological conditions of human and mouse (tumor; autoimmune disease; infectious diseases; transplantation immunity, etc.). The main overview and progress of the research objects, methods, and characteristics of dual TCR T cells.

## Data Availability

The data used to paint are available in the NCBI repository https://www.ncbi.nlm.nih.gov/geo/query/acc.cgi?acc=GSE140133; https://www.ncbi.nlm.nih.gov/geo/query/acc.cgi?acc=GSE168944.
