# Supplementary material for: Proportion, Function, and Generation of Dual Receptor Lymphocytes
Source: J Immunol Res. 2026 Feb 14;2026:5510858. doi: 10.1155/jimr/5510858 (PMC13140939; doi:10.1155/jimr/5510858)
Supplement: Supplementary file 1 — Supporting Information Figure S1. Based on the composition and arrangement order of VDJC genes in the murine TRB locus, we constructed a schematic diagram detailing the sequential processes of VDJ recombination on a single mouse chromosome under the framework of classical VDJ recombination rules. This includes the inversion recombination involving the inverted V31 gene, the loop‐out recombination involving the forward Vx gene, as well as the canonical sequential order of D‐J rearrangement followed by V‐DJ rearrangement. Furthermore, we propose a potential mechanism by which two rounds of functional VDJ recombination and subsequent transcription can be accomplished on a single chromosome in accordance with classical recombination rules. Figure S2. Example Visualization of the Results from the 1985 Dual BCR Study (Feddersen and Van Ness [35], Proceedings of the National Academy of Sciences, 1985). Figure S3. Example Visualization of the Results from the 1988 Dual TCR Study (Triebel et al. [11], Journal of Immunology, 1988). Figure S4. Example Visualization of the Results from the 1995 TCR+BCR+ Study (Hanawa et al. [127], Leukemia & Lymphoma, 1995). Table S1‐1. In physiological conditions of humans and mice: The main overview and progress of the research objects, methods, and characteristics of dual BCR B cells/TCR + BCR + lymphocyte. Table S1‐2. In pathological conditions of humans and mice (B‐cell associated tumor; autoimmune diseases, etc.): The main overview and progress of the research objects, methods, and characteristics of dual BCR B cells/TCR + BCR + lymphocyte. Table S2‐1. In physiological conditions of humans and mice: The main overview and progress of the research objects, methods, and characteristics of dual TCR T cells. Table S2‐2. In pathological conditions of human and mouse (tumor; autoimmune disease; infectious diseases; transplantation immunity, etc.). The main overview and progress of the research objects, methods, and characteristics of dual TCR T [file JIMR-2026-5510858-s001.docx]

**Supplement Material**

**Supplement Figure : Sup Fig 1; Sup Fig 2; Sup Fig 3; Sup Fig 4.**

**
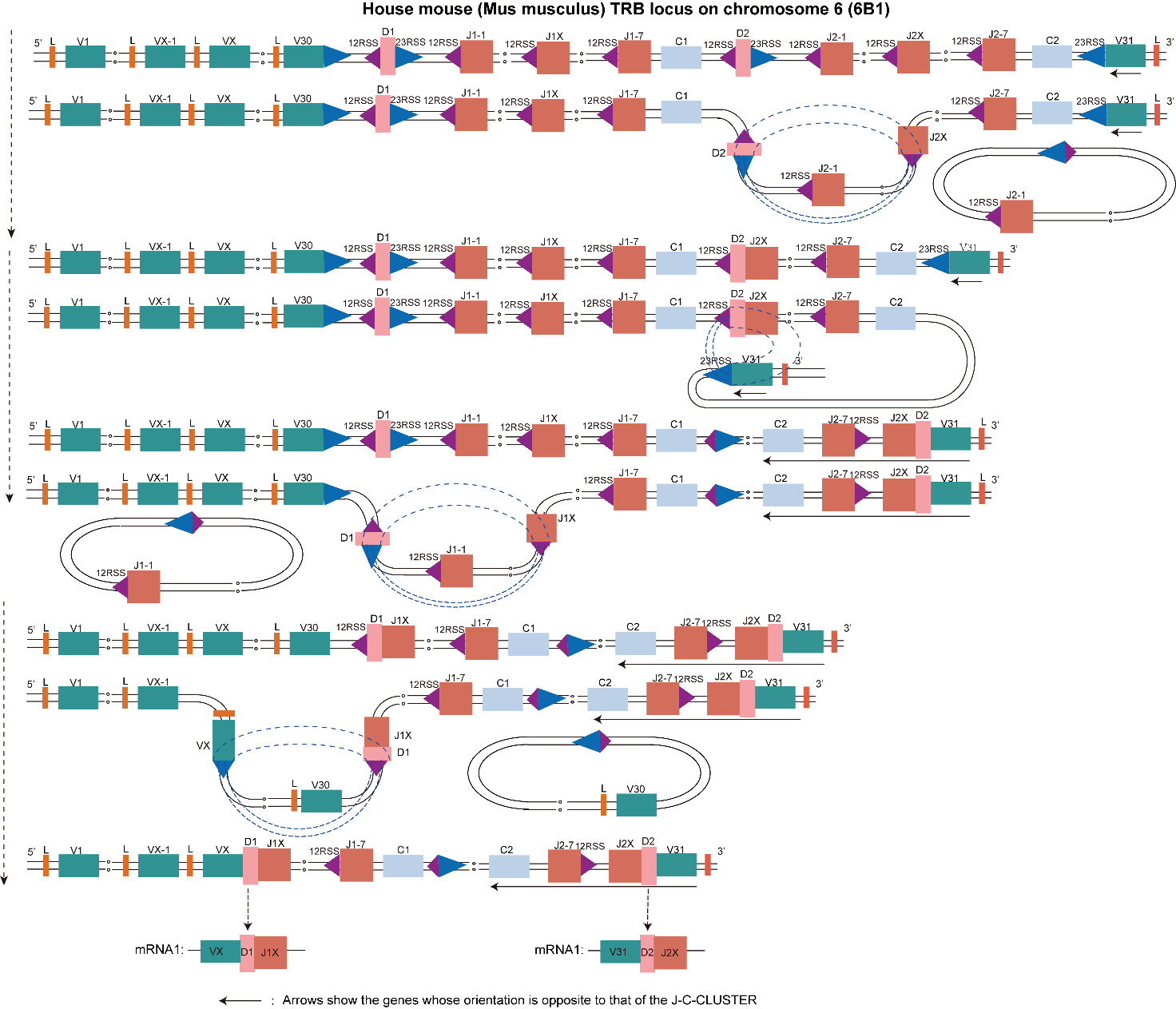
**

**Sup Fig 1.** Based on the composition and arrangement order of VDJC genes in the murine TRB locus, we constructed a schematic diagram detailing the sequential processes of VDJ recombination on a single mouse chromosome under the framework of classical VDJ recombination rules. This includes the ****inversion recombination**** involving the inverted V31 gene, the ****loop-out recombination**** involving the forward Vx gene, as well as the canonical sequential order of D-J rearrangement followed by V-DJ rearrangement. Furthermore, we propose a potential mechanism by which two rounds of functional VDJ recombination and subsequent transcription can be accomplished on a single chromosome in accordance with classical recombination rules.


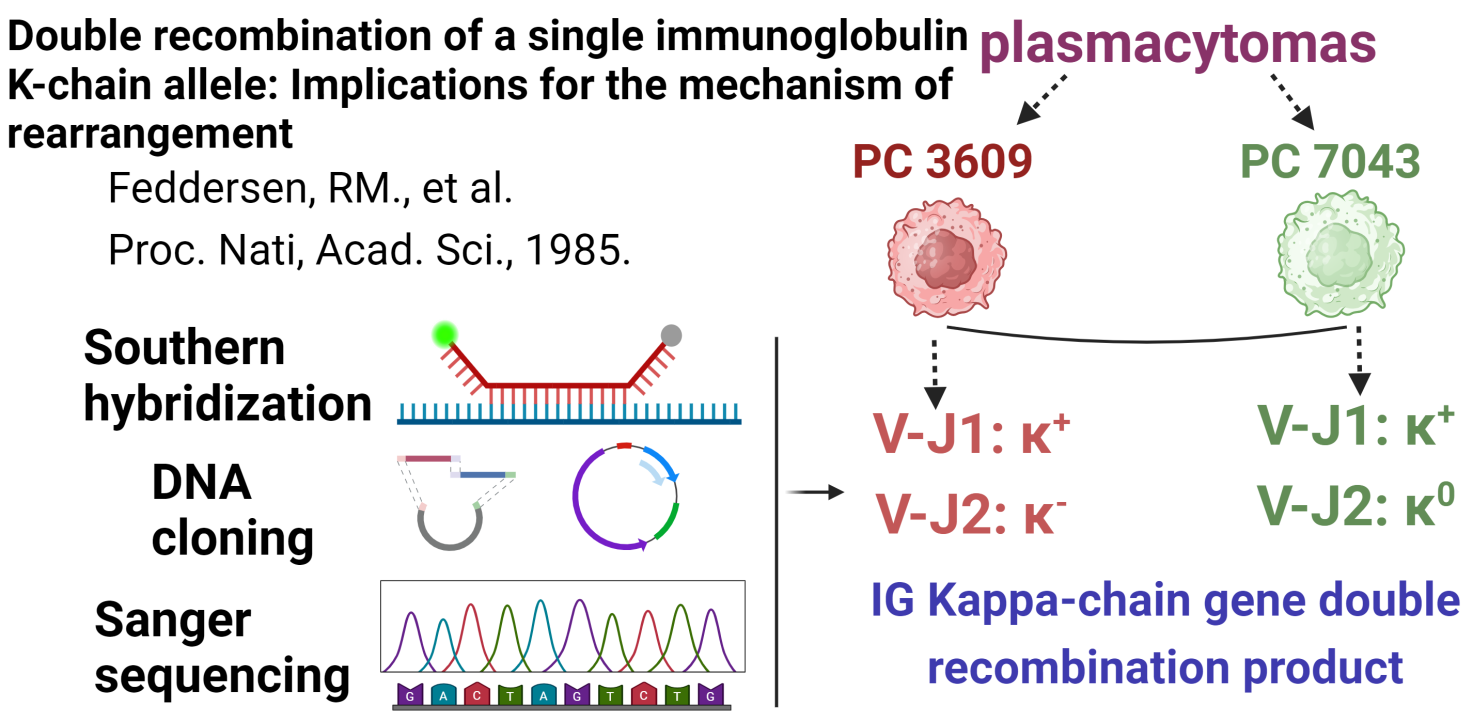


**Sup Fig 2.** Example Visualization of the Results from the 1985 Dual BCR Study (Feddersen, R.M., et al., Proceedings of the National Academy of Sciences, 1985).


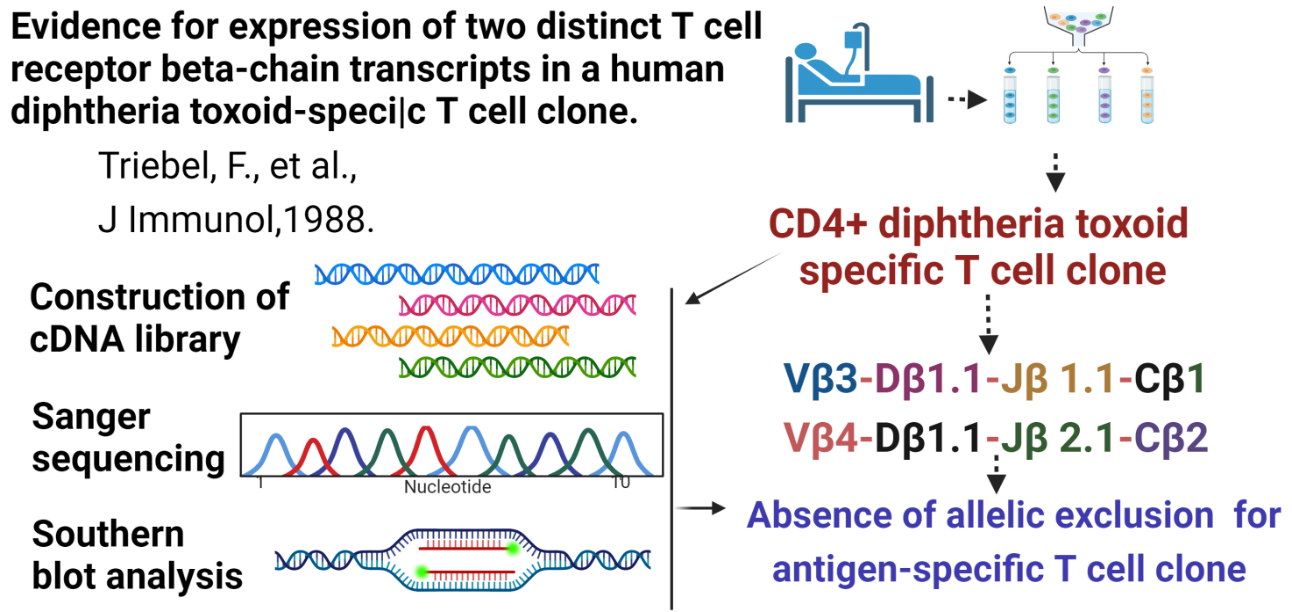


**Sup Fig 3.** Example Visualization of the Results from the 1988 Dual TCR Study (Triebel, F., et al., Journal of Immunology, 1988).


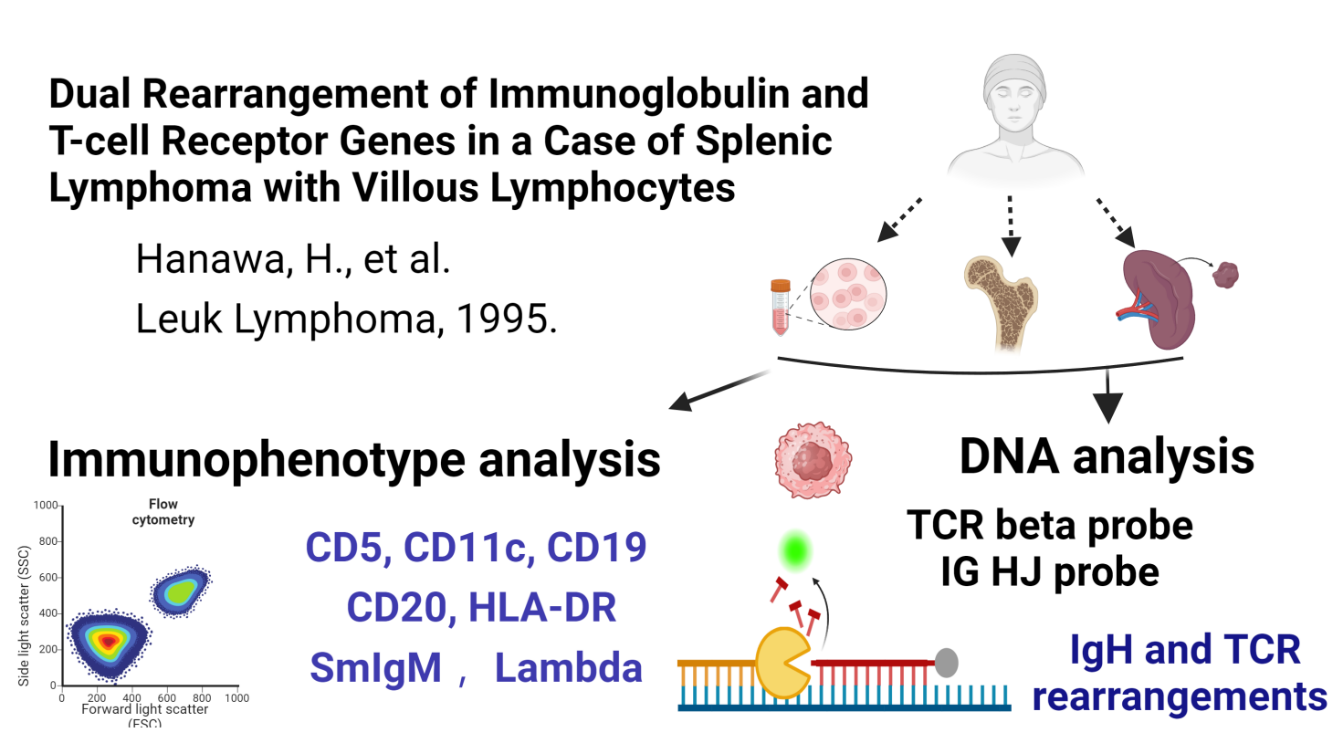


**Sup Fig 4.** Example Visualization of the Results from the 1995 TCR+BCR Study (Hanawa, H., et al., Leukemia & Lymphoma, 1995).

**Supplement Table: Sup Tab 1-1; Sup Tab 1-2; Sup Tab2-1; Sup Tab 2-2**

**Sup Tab 1-1.** **In physiological conditions of humans and mice:** The main overview and progress of the research objects, methods, and characteristics of dual BCR B cells/TCR+BCR+lymphocyte.

| Time | Study object，sample | Detection methods | Proportion, function, composition characteristics, etc. | References |
| --- | --- | --- | --- | --- |
| 1961 | Immunized of rats with flagella or flagellin，Isolate single cells from lymph nodes. | The cells had been incubated singly in microdrops, and anti-H antibody was detected by immobilization of motile bacteria. | Out of 1333 cells from the hind foot pads of rats immunized with flagella, 455 produced either anti-H or anti-O antibody; 4 produced both;out of 360 cells from rats hind foot pads immunized by flagellin, 213 produced either anti-H or anti-0 antibody; 3 produced both.（dual BCR plasma cell） | Makela, O. et al., Study of antibody-producing capacity of single cells by bacterial adherence and immobilization. J Immunol, 1961. 87: p. 457-63. |
| 1995 | 5 healthy people，peripheral blood cells | ELISA, Flow cytometry，southern blot analysis. | The proportion of κ^+^ λ^+^ B cells, which are capable of expressing both κ and λ immunoglobulin chains, was 0.2-0.5% in the peripheral blood B cells of five healthy donors.（dual BCR--Hκλ B cells) | Giachino, C., et al., .kappa+lambda+ dual receptor B cells are present in the human peripheral repertoire. J Exp Med, 1995. 181(3): p. 1245-50. |
| 1998 | Healthy C57BL/6 mice and lambda5-deficient mice(Lambda5 knockout mice；Pathological model), spleen. | Flow cytometry | 2%-4% of B cells carried two functional V_H_-D_H_-J_H_ rearrangements（dual BCR--HHκ/λ B cells) | en Boekel, E., et al., Precursor B cells showing H chain allelic inclusion display allelic exclusion at the level of pre-B cell receptor surface expression. Immunity, 1998. 8(2): p. 199-207. |
| 2001 | Igκ ^m/h^ mice (Transgenic mice containing both human and mouse Igk genes; Pathological model) bone marrow and spleen. | Flow cytometry，PCR, sanger sequencing. | The B cell content of dual BCR was 5%, and only 1.5% of B cells in Igk ^m/h^ mice expressed dual Igk chains（dual BCR B cells) （dual BCR--Hκλ B cells). | Casellas, R., et al., Contribution of receptor editing to the antibody repertoire. Science, 2001. 291(5508): p. 1541-4. |
| 2004 | B cell nuclear transfer mouse (Mice generated through nuclear transfer with specific IgL allele genes.)，bone marrow, spleen, peripheral blood cells. | ELISA, Flow cytometry. | Identified two functional IgL gene rearrangements and receptor expressions in the B cell nuclear transfer mouse.with cells expressing two different receptors（dual BCR B cells). | Gerdes, T. et al., Autoreactivity and allelic inclusion in a B cell nuclear transfer mouse. Nat Immunol, 2004. 5(12): p. 1282-7. |
| 2005 | Peripheral blood B cells from three healthy people. | Single-cell RNA sequencing | In peripheral blood from healthy donors 1 and 2, the researchers detected V_L_ allele inclusion at approximately 0.4% of the V_H_ cluster, and 14 allele containing antibodies were detected in donor 3（dual BCR B cells). | DeKosky, B.J., et al., In-depth determination and analysis of the human paired heavy- and light-chain antibody repertoire. Nat Med, 2015. 21(1): p. 86-91. |
| 2005 | 3-83Igi H-2d (Transgenic mice carrying the 3-83Ig gene) B1-8/3-83Igi H-2b (Transgenic mice carrying the B1-8 and 3-83 genes. Pathological model);BALB/c or CB17, Bone marrow and spleen. | ELISA, Flow cytometry. | A high proportion (30-40%) of allelic inclusion rearranged B cells in nonautoreactive mice 3-83Igi H-2d, dual antibody expressing mice B1-8/3-83Igi H-2b, as well as wild type BALB/c or CB17 mice（dual BCR B cells). | Liu, S., et al., Receptor editing can lead to allelic inclusion and development of B cells that retain antibodies reacting with high avidity autoantigens. J Immunol, 2005. 175(8): p. 5067-76. |
| 2005 | C57BL/6mice，bone marrow，peritonem, Spleen lymphocytes. | Flow cytometry | κ^+^ λ^+^ cells in 2-6 months C57BL/6 mice was: bone marrow 0.12±0.06, spleen 0.96±0.29 and peritoneum 1.66±0.82（dual BCR--Hκλ B cells). | Rezanka, L.J., et al., Dual isotype expressing B cells [kappa(+)/lambda(+)] arise during the ontogeny of B cells in the bone marrow of normal nontransgenic mice. Cell Immunol, 2005. 238(1): p. 38-48. |
| 2007 | Igκ m/h mice（Transgenic mice containing both human and mouse Igk genes；Pathological model）, spleen. | Gene targeting technology,  Immunohistochemistry,  Flow Cytometry. | 10% of mature B cells expressed both Igk alleles（dual BCR--Hκκ B cells). | Casellas, R., et al., Igkappa allelic inclusion is a consequence of receptor editing. J Exp Med, 2007. 204(1): p. 153-60. |
| 2007 | Igκ m/h mice（Transgenic mice containing both human and mouse Igk genes；Pathological model）, spleen. | Flow cytometry,  Immunohistochemistry. | B cells expressing two different Ig kappa chains accounted for 1.4-3% of all B cells（dual BCR--Hκκ B cells).. | Velez, M.G., et al., Ig allotypic inclusion does not prevent B cell development or response. J Immunol, 2007. 179(2): p. 1049-57. |
| 2019 | Five healthy donors，peripheral blood mononuclear cells. | Chromium single cell sequencing, Sanger sequencing. | Two V_H_DJ_H_ (5.90-8.71%) or V_K_J_K_ (9.71-13.07%) V_λ_J_λ_ (12.69-20.07%) were observed in thousands of individual B cells, and even more than two V_H_DJ_H_ (0.16-0.42%) or V_K_J_K_ (0.21- 0.49%), V_λ_J_λ_ (0.30-0.58%) recombination patterns. Each Ig class displays a unique V_H_DJ_H_ recombination pattern in a single B cell（dual BCR B cells). | Shi, Z., et al., More than one antibody of individual B cells revealed by single-cell immune profiling. Cell Discov, 2019. 5: p. 64. |
| 2023 | 9 healthy volunteers and 4 healthy mice,Bone marrow and peripheral blood. | Single-cell BCR sequencing | Human bone marrow and peripheral blood contain about 10% dual (or multiple) BCR B cells, while in mouse peripheral blood and bone marrow memory B cells this proportion reaches around 20%（dual BCR B cells).. | Zhu, L., et al., scBCR-seq revealed a special and novel IG H&L V(D)J allelic inclusion rearrangement and the high proportion dual BCR expressing B cells. Cell Mol Life Sci, 2023. 80(11): p. 319. |
| 2024 | Mouse peritoneal cavity and peripheral blood from healthy volunteers | scRNA + BCR-seq | A high proportion of B cells expressing dual receptors, predominantly dual κ-chains, was observed in the mouse peritoneal cavity. Furthermore, the frequency of dual-BCR B cells was increased in aged mice | [Huifang Wang](https://pubmed.ncbi.nlm.nih.gov/?term=Wang+H&cauthor_id=39732722) , , et al., scRNA + BCR-seq identifies proportions and characteristics of dual BCR B cells in the peritoneal cavity of mice and peripheral blood of healthy human donors across different ages. Immun Ageing. 2024 Dec 28;21(1):90. |

**Sup Tab 1-2. In pathological conditions of humans and mice (B-cell associated tumor; autoimmune diseases, etc.):** The main overview and progress of the research objects, methods, and characteristics of dual BCR B cells/TCR+BCR+lymphocyte.

| Time | Study object，sample | Detection methods | Proportion, function, composition characteristics, etc. | References |
| --- | --- | --- | --- | --- |
| 1985 | Two different plasma cell tumors，PC 7043 and PC 3609 （Tumor) | Southern hybridization，Sanger sequencing. | In PC3609, one allele was functional (κ^+^) while the other was non-functional (κ^-^). whereas in PC 7043 one of the K-chain alleles remains in the germ-line configuration (κ^O^) and the other has rearranged productively (κ^+^).（dual BCR--Hκκ B cells) | Feddersen, R.M, et al., Double recombination of a single immunoglobulin kappa-chain allele: implications for the mechanism of rearrangement. Proc Natl Acad Sci U S A, 1985. 82(14): p. 4793-7. |
| 1988 | One case of CLL patient，peripheral blood B cell（Tumor) | Southern hybridization analysis, immunoglobulin gene rearrangements. | The patient showed monoclonal rearrangements of constant μ、κ, and λ genes, dual rearrangements of IGLκ and IGLλ genes, and 85% of kappa-positive cells and 5% of lambda-positive cells.（dual BCR--Hκλ B cells) | Peltomäki, P., et al., Immunoglobulin kappa and lambda light chain dual genotype rearrangement in a patient with kappa-secreting B-CLL. Eur J Cancer Clin Oncol, 1988. 24(7): p. 1233-8. |
| 1995 | Splenic lymphoma with villous lymphocytes(SLVL) patient，Peripheral blood cells（Tumor) | Southern blot | Immunological analysis of peripheral leukocytes revealed B cell and T cell phenotypes (CD5, CD19, CD20, HLA-DR, SmIgM, and λ positive), and DNA analysis revealed dual rearrangement of immunoglobulin heavy chain genes and T cell receptor βgenes. （TCR+BCR+ lymphocytes） | Hanawa, H., et al., Dual rearrangement of immunoglobulin and T-cell receptor genes in a case of splenic lymphoma with villous lymphocytes. Leuk Lymphoma, 1995. 18(3-4): p. 357-60. |
| 1998 | A case of chronic lymphocytic leukemia，Peripheral blood cells（Tumor). | Flow cytometry | Presence of a unique kappa/lambda dual positive (κ^+^ λ^+^:23%) tumor cell population（dual BCR--Hκλ B cells). | Kawada, H., et al., A novel variant of B-lymphoid leukemia expressing kappa/lambda light chains. Acta Haematol, 1998. 100(1): p. 54-6. |
| 2000 | Plasmacytoma: SIPC3282； SIPC3301；MOPC104E（Tumor). | ELISA, RT-PCR | The plasmacytoma SIPC3282 secretes both Igk and Igλ, with Igk levels significantly higher than Igλ. SIPC3301 and MOPC104E expressed higher levels of Igλ than Igk（dual BCR--Hκλ B cells).. | Diaw, L., et al., Double producers of kappa and lambda define a subset of B cells in mouse plasmacytomas. Mol Immunol, 2000. 37(12-13): p. 775-81. |
| 2000 | 1. Ig-Tg mice (Dual Ig transgenic mice; Pathological model), spleen（Autoimmune diseases）. | Flow cytometry | initial B cells in the spleens of D-Ig-Tg mice expressed two types of BCR, but the majority of activated B cells expressed only one type of BCR（dual BCR B cells). | Rosado, M.M,et al., B cell positive selection by self antigens and counter-selection of dual B cell receptor cells in the peripheral B cell pools. Eur J Immunol, 2000. 30(8): p. 2181-90. |
| 2002 | 398 cases of lymphoma patients，peripheral blood cells（Tumor). | Microdissection of single cells, Whole genome amplification. | In 398 cases of lymphoma, 13% of B cell lymphoma patients exhibited a dual genotype, characterized by the presence of both T cell receptor gamma gene rearrangement and immunoglobulin heavy chain gene rearrangement（TCR+BCR+ lymphocytes）. | Vergier, B., et al., Combined analysis of T cell receptor gamma and immunoglobulin heavy chain gene rearrangements at the single-cell level in lymphomas with dual genotype. J Pathol, 2002. 198(2): p. 171-80. |
| 2004 | LamH×V8R H+L dual Tg mice (Transgenic mice carrying LamH-Cμ H chain and functional Vκ8Jκ5 L chain; Pathological model)，LamH × V8R/V8R H + LL triple-Tg mice（Transgenic mice carrying LamH H chain transgene and homozygous for V8R transgene）,spleen.（Autoimmune diseases） | Flow cytometry, ELISA，Immunofluorescence. | LamHV8R/V8R triple Tg mice, expressed Tg and endogenous H chain at the same time,with 2.6–3.2% background staining in L-Tg and WT mice，The V8R transgene used J5, indicating secondary V-J rearrangement on non-targeted allele. The researchers obtained thirty-six clones yielded L chain sequences, of which 17 were the V8R Tg L chain. The other 19 L chains were endogenous in origin（dual BCR--Hκκ B cells).. | Brady, G.F., et al., Kappa editing rescues autoreactive B cells destined for deletion in mice transgenic for a dual specific anti-laminin Ig. J Immunol, 2004. 172(9): p. 5313-21. |
| 2006 | Primary cutaneous lymphoproliferative disorders，diseased tissue（Tumor) | PCR，Southern blot，Immunohistochemistry. | 15 cases (about 1%) had both TCR and IgH gene monoclonal rearrangements among about 1200 cases of primary skin TCR and IgH rearrangement proliferative diseases（TCR+BCR+ lymphocytes）. | Kazakov, D.V., et al., Primary cutaneous lymphoproliferative disorders with dual lineage rearrangement. Am J Dermatopathol, 2006. 28(5): p. 399-409. |
| 2007 | A case of B-cell lymphoma/sorted bone marrow cells（Tumor). | Flow cytometry | The expression of dual immunoglobulin light chains in a case of B-cell lymphoma.. Notably, the lymphoma cells were positive for both Igκ (97%) and Igλ (89%) based on a CD19^+^ gate strategy（dual BCR--Hκλ B cells).. | Fujiwara, T., et al., Aggressive B-cell lymphoma with dual surface immunoglobulin light-chain expression. Intern Med, 2007. 46(17): p. 1458-61. |
| 2008 | Anti-dnaH chain transgenic mouse B6.56R（Transgenic C57/BL/6 mice carrying the 56R gene; Pathological model）and BALB/c.56R（Transgenic BALB/c mice carrying the 56R gene,Pathological model）, peripheral blood B cells（Autoimmune diseases）. | Flow cytometry | Screened 364 cells which included 56R and 56R B cells from B6.56R and BALB/ c.56R mice, and identified 2 different in-frame dual λ cells in 48 cells (12.9%)（dual BCR--Hκκ B cells).. | Witsch, E.Jet al., Allelic and isotypic light chain inclusion in peripheral B cells from anti-DNA antibody transgenic C57BL/6 and BALB/c mice. J Immunol, 2008. 180(6): p. 3708-18. |
| 2011 | Angioimmunoblastic T-cell lymphoma，tumor tissue（Tumor). | Southern blot | Southern blotting analysis showed simultaneous rearrangement of TCR-Cβ1 and IGH-JH genes（TCR+BCR+ lymphocytes）.. | Aung, N.Y., et al., Angioimmunoblastic T-cell lymphoma with dual genotype of TCR and IgH genes. Pathol Res Pract, 2011. 207(5): p. 317-21. |
| 2012 | Healthy C57BL/6 mice and autoimmune MRL/lpr mice（Autoimmune disease model; Pathological model）, spleen.（Autoimmune diseases）. | Flow cytometry,  Immunofluorescence (IF). | Two types of Ig light or heavy chain B cells were found. In the spleen of MRL/lpr mice, B cells expressing dual k accounted for half of the plasmablast and memory B cells（dual BCR B cells)... | Fournier, E.M., et al., Dual-reactive B cells are autoreactive and highly enriched in the plasmablast and memory B cell subsets of autoimmune mice. J Exp Med, 2012. 209(10): p. 1797-812. |
| 2013 | hCk/mCk（Transgenic mice containing both human and mouse Igk genesl）, NZB/NZW,D42H NZB/NZW（NZB/NZW mice carrying the D42H gene）, Anti-DNA autoreactive B cells（Autoimmune diseases）. | Flow cytometry,  Single-cell and hybridoma analysis. | Approximately 4% of B cells in hCk/mCk (B6xBALB/c) F1 mice expressed both human and mouse Cκ light chains, while D42 transgenic NZB/NZW mice had a higher proportion of dual L-chain B cells (13-40%)（dual BCR B cells). | Makdasi, E,et al., L chain allelic inclusion does not increase autoreactivity in lupus-prone New Zealand Black/New Zealand White mice. J Immunol, 2013. 190(4): p. 1472-80. |
| 2013 | A patient with splenic marginal zone lymphoma，peripheral blood cells（Autoimmune diseases）.. | Flow cytometry,  Electron microscopy. | 99% of circulating lymphoma cells demonstrated dual expression of both kappa and lambda light chains（dual BCR B cells).. | Montague, N., et al., Platelet satellitism and dual surface immunoglobulin light-chain expression in circulating splenic marginal zone lymphoma cells. Ann Diagn Pathol, 2013. 17(1): p. 117-22. |
| 2015 | Patients with systemic lupus erythematosus,peripheral blood B cells（Autoimmune diseases）. | Flow cytometry | Approximately 54% of SLE patients were positive for Igκ and Igλ light chains in CD19+ B cells（dual BCR--Hκλ B cells).. | Fraser, L.D., et al., Immunoglobulin light chain allelic inclusion in systemic lupus erythematosus. Eur J Immunol, 2015. 45(8): p. 2409-19. |
| 2015 | Plasma cell neoplasm patient，bone marrow（Tumor).. | Flow cytometry,  electrophoretic analysis. | Up to 2% of the plasma cell neoplasm considered being biclonal based on electrophoretic analysis, characterized by secretion of paraprotein with two distinct heavy chains or light chains（dual BCR--Hκλ B cells)... | Jiwani, S.,et al., Biphenotypic plasma cell myeloma: two cases of plasma cell neoplasm with a coexpression of kappa and lambda light chains. Int J Clin Exp Pathol, 2015. 8(7): p. 8536-44. |
| 2016 | 55 canine cases with centroblastic lymphoma, lymph node（Tumor).. | Flowcytometry,  polymerase chain reaction (PCR). | In two cases the PCR techniques confirmed the presence of simultaneous dual gene rearrangements of the BCR and TCR receptor, and the results of this study reveal that co-expression of both B-cell and T-cell markers in these cells（TCR+BCR+ lymphocytes）... | Pawlak, A., et al., Immunophenotypic characterization of canine malignant lymphoma: a retrospective study of cases diagnosed in Poland Lower Silesia, over the period 2011-2013. Veterinary and comparative oncology, 2016. 14 Suppl 1: p. 52-60. |
| 2017 | A case of bladder cancer，sentinel lymph node （Tumor) | Flow cytometry | A rare Igλ^+^Igκ^+^ B cell population was found to be increased in tumor associated tissues, and present in activated B cell populations, as well as among switched memory B lymphocytes. which do not undergo somatic hypermutation and receptor editing（dual BCR--Hκλ B cells).. | Zirakzadeh, A.A. B Lymphocytes in solid human malignancies. 2017. |
| 2018 | Three patients with plasma cell myeloma，bone marrow（Tumor). | Flow cytometry,  Immunohistochemistry,  Insituhybridization. | Flow cytometry detected abnormal plasma cells expressing CD56 and kappa and lambda light chains simultaneously, and IHC and ISH found that plasma cells strongly expressed both kappa and lambda（dual BCR--Hκλ B cells)... | Jiang, A.S., et al., Plasma cell myeloma with dual expression of kappa and lambda light chains. Int J Clin Exp Pathol, 2018. 11(9): p. 4718-4723. |
| 2018 | autoimmune mice (Igκm/h) （Transgenic mice containing both human and mouse Igk genes；Pathological model）,spleen（Autoimmune diseases）. | RNA-seq | The number of dual BCR B cells in autoimmune mice (Igκ^m/h^) was higher than that in healthy mice and humans (3%)（dual BCR B cells).. | Sang, A., et al., Innate and adaptive signals enhance differentiation and expansion of dual-antibody autoreactive B cells in lupus. Nat Commun, 2018. 9(1): p. 3973. |
| 2019 | Three subjects with type 1 diabetes (T1D)，peripheral blood cells（Autoimmune diseases）. | Flow cytometry | Identified approximately 2.2% of "lymphocytes" with dual expression of TCR and BCR by flow cytometry in three subjects with type 1 diabetes (T1D), and similarly found 0.4% of cells with both TCR and BCR receptors on the surface in healthy controls（TCR+BCR+ lymphocytes）. | Ahmed, R., et al., A Public BCR Present in a Unique Dual-Receptor-Expressing Lymphocyte from Type 1 Diabetes Patients Encodes a Potent T Cell Autoantigen. Cell, 2019. 177(6): p. 1583-1599.e16. |
| 2021 | A case of solitary plasmacytoma of bone，tumor tissue（Tumor) | Flow cytometry | Approximately 96% of these tumor cells co-expressed cytoplasmic kappa and lambda light chain based on a CD38 positive gate strategy（dual BCR--Hκλ B cells)... | Matsuoka, R., et al., A case of solitary plasmacytoma of bone showing co-expression of both immunoglobulin light chains. Eur J Med Res, 2021. 26(1): p. 148. |
| 2022 | 18 SLE patients,peripheral blood samples（Autoimmune diseases）. | Flow cytometry, Microscope. | About a quarter of SLE patients exhibited a higher frequency of dual receptor B cells, the elevation in k^+^ λ^+^ B cells resulted from the decoration of VH4-34 ( k and λ) autoantibodies（dual BCR B cells). | Peterson, J.N., et al., Elevated Detection of Dual Antibody B Cells Identifies Lupus Patients With B Cell-Reactive VH4-34 Autoantibodies. Front Immunol, 2022. 13: p. 795209. |
| 2023 | Patients with diffuse large B-cell lymphoma，tumor tissue（Tumor). | PCR Immunohistochemistry, polymerase chain reaction. | In primary nodal unclassifiable CD20 negative diffuse large B-cell lymphoma that did not express CD20 in immunohistochemical staining and showed a dual rearrangement of both Igκ and TCR genes（TCR+BCR+ lymphocytes）. | Hadžisejdić, I., et al., Primary Nodal Unclassifiable CD20 Negative Diffuse Large B-cell Lymphoma With Dual IgK and TCR Gene Rearrangement: A Diagnostic Challenge. Clinical Pathology, 2023. 16. |

**Sup Tab 2-1. In physiological conditions of humans and mice:** The main overview and progress of the research objects, methods, and characteristics of dual TCR T cells

| Time | Study object, sample | Detection methods | Proportion, function, composition characteristics, etc | References |
| --- | --- | --- | --- | --- |
| 1988 | B10.A mouse（Physiological model），spleen monoclonal T cells. | Southern blot, Northern blot, Sanger sequencing. | B10.A mouse spleen monoclonal T cells showed dual functional V α gene rearrangements (both transcribed into 1.5 kb mRNA), and the dual α chains were combined with β respectively. However, only one can simultaneously recognize allogeneic MHC and self-MHC-limiting antigen(dual TCR--*ααβ* T cells). | Malissen, M., et al., A T cell clone expresses two T cell receptor alpha genes but uses one alpha beta heterodimer for allorecognition and self MHC-restricted antigen recognition. Cell, 1988. 55(1): p. 49-59. |
| 1988 | Pigeon cytochrome C immunized B10.S(9R),B10.A（bone marrow chimeric mouse, Pathological model），cloned T cells. | Southern blot, radioimmunoprecipitation，Gel electrophoresis. | Two different rearrangement of Vβ1 gene fragments (14.0kb and 7.2 kb) in C.D2 cloned T cellsd(dual TCR--*αββ* T cells)... | Matis, L.A., et al., Expression of two distinct T cell receptor alpha/beta heterodimers by an antigen-specific T cell clone. J Exp Med, 1988. 168(6): p. 2379-84. |
| 1989 | KLH-pnmed C3H mice（Physiological model）, I-AK autoreactive helper T cell clone MS202. | Sanger sequencing, Southern blot, RNA blot, Immunoprecipitation, Gel electrophoresis. | Dual α chain cDNA clones showed in-frame rearrangements and that only one α chain polypeptides was expressed on the cell surface(dual TCR--*ααβ* T cells).. | Furutani, M., et al., Post-transcriptional allelic exclusion of two functionally rearranged T cell receptor alpha genes. Int Immunol, 1989. 1(3): p. 281-8. |
| 1991 | Transgenic mice（TCR Vβ2 and TCRVβ8.2 dual transgenic mice, Physiological model）,thymus and lymph nodes T cells. | Flow Cytometry | Most of the T cells could simultaneously express two TCRβ chains. These T cells could be selected by the thymus and their TCR could be transduced and activated in vitro(dual TCR--*αββ* T cells).. | van Meerwijk, J.P., et al., Allelic exclusion at DNA rearrangement level is required to prevent coexpression of two distinct T cell receptor beta genes. J Exp Med, 1991. 174(4): p. 815-9. |
| 1991 | CBA/J mice（Physiological model）,BM3.3 CD8+T cells. | Southern blot，Northern blot，Sanger sequencing. | H-2Kb specific cytotoxicity CD8+T cell clone BM3.3 can carry dual effective TCRs α Gene rearrangement, but only a single TCR can be detected on its cell surface(dual TCR--*ααβ* T cells)... | Couez, D., et al., Each of the two productive T cell receptor alpha-gene rearrangements found in both the A10 and BM 3.3 T cell clones give rise to an alpha chain which can contribute to the constitution of a surface-expressed alpha beta dimer. Int Immunol, 1991. 3(7): p. 719-29. |
| 1992 | Transgenic mice（Transgenic α (T3.70) and β (F23.1) TCR chains, Physiological model）, thymic T cells. | Flow Cytometry | Thymic T cells would try different TCRαβ combinations to bind to thymic MHC ligands, and the rearrangement of α chains would stop only after positive selection(dual TCR T cells).. | Borgulya, P., et al., Exclusion and inclusion of alpha and beta T cell receptor alleles. Cell, 1992. 69(3): p. 529-37. |
| 1993 | Normal donor PBMCs | Flow Cytometry | Used three anti-human Vα2, Vα12 and Vα24 antibodies to stain normal donor PBMCs with a combination of two Vα antibodies, 0.01% to 0.1% of cells bound to the two antibodies were detected. When dual positive cells were selected and cloned, 1/3 to 1/2 of the clones stably expressed Va chain.(dual TCR--*ααβ* T cells). | Padovan, E., et al., Expression of two T cell receptor alpha chains: dual receptor T cells. Science, 1993. 262(5132): p. 422-4. |
| 1993 | Transgenic mice（TCR β transgenic mice, Physiological model）, lymph nodes CD8 + T cells. | Flow Cytometry | CD8 + T cells in the lymph nodes of TCR β transgenic mice expressed two different TCR α chain clones, one was the product of α trans-gene, and the other was the product of endogenous rearrangement(dual TCR--*ααβ* T cells).. | Heath, W.R,et al., Expression of two alpha chains on the surface of T cells in T cell receptor transgenic mice. J Exp Med, 1993. 178(5): p. 1807-11. |
| 1993 | TCR transgenic mice | Flow Cytometry，Sanger sequencing. | TCR α gene rearrangement continues even after surface expression on TCR α/β heterodimers until the recombination process is positively selected to stop or until cell death occurs(dual TCR T cells). | H T Petrie ,,et al., .Multiple rearrangements in T cell receptor alpha chain genes maximize the production of useful thymocytes.J Exp Med. 1993 Aug 1;178(2):615-22. doi: 10.1084/jem.178.2.615. |
| 1995 | B10.BR and C57BL/6 (B6) mice（Physiological model）, lymph node T cells | Flow Cytometry | The frequency of two functional α chains in normal mouse lymph node T cells was 7-21%, with an average of 15% (dual TCR--*ααβ* T cells). | Heath, W.R., et al., Expression of two T cell receptor alpha chains on the surface of normal murine T cells. Eur J Immunol, 1995. 25(6): p. 1617-23. |
| 1995 | B10.BR mice（Vβ3-Dβ2-Jβ2.5 transgenic mice, Physiological model）, C57BL/6 mice（TCR α-hemizygous mice, Physiological model）, Immature and mature thymus T cells | Flow Cytometry | Dual V α chain was common in immature T cells and in TCR alpha-hemizygous mice not found, suggesting that cell surface expression of dual α chain is caused by the lack of allelic exclusion in immature thymocytes (dual TCR--*ααβ* T cells).. | Alam, S.M.,et al., Allelic exclusion of mouse T cell receptor alpha chains occurs at the time of thymocyte TCR up-regulation. Immunity, 1995. 3(4): p. 449-58. |
| 1995 | Healthy, PBL T cells, | Flow Cytometry | Nearly 1% of T cells in human PBL in healthy individuals express two types of antigen responsive TCR β chains(dual TCR--*αββ* T cells)... | Davodeau, F., et al., Dual T cell receptor beta chain expression on human T lymphocytes. J Exp Med, 1995. 181(4): p. 1391-8. |
| 1995 | human peripheral blood T cells | Flow Cytometry | About 1% of human peripheral blood T cells express dual Vβs, and dual TCRβ expressing T cells existed in both CD45R0+ and CD45R0- subsetss(dual TCR--*αββ* T cells). | Padovan, E., et al., Normal T lymphocytes can express two different T cell receptor beta chains: implications for the mechanism of allelic exclusion. J Exp Med, 1995. 181(4): p. 1587-91. |
| 1995 | Transgenic mice（TCR Vβ8.2 transgenic mice, Physiological model）, thymus, spleen, lymph node T cells. | Flow Cytometry | On most T cells of TCR transgenic mice bearing two TCRs, only one is specific for peptides bound to self-MHC molecules. The expression of dual TCR usually does not confer reactivity to two unrelated antigens(dual TCR T cells). | Hardardottir, F.,et al., T cells with two functional antigen-specific receptors. Proc Natl Acad Sci U S A, 1995. 92(2): p. 354-8. |
| 1995 | TCR transgenic mice（Vβ3/Vα11, Physiological model）, thymus, spleen T cells. | Flow Cytometry | 29±2% of peripheral T cells expressed dual V β chain in TCR transgenic mice, and dual V β chain expressing cells increased significantly in the periphery with age, with 9±3.7% of mature T cells expressing dual β chain in normal 9-12 months old mice(dual TCR--*αββ* T cells).. | Balomenos, D., et al., Incomplete T cell receptor V beta allelic exclusion and dual V beta-expressing cells. J Immunol, 1995. 155(7): p. 3308-12. |
| 1996 | BALB/c mice（TCRαβ transgenic mice, Physiological model），spleen CD4+T cells. | Flow Cytometry | About 5-7% of spleen CD4+T cells expressed exogenous β chain and endogenous TCRβ chain, and dual β chain had two different specificities.(dual TCR--*αββ* T cells).. | Munthe, L.A., et al., T cells with two Tcr beta chains and reactivity to both MHC/idiotypic peptide and superantigen. Cell Immunol, 1996. 170(2): p. 283-90. |
| 1997 | TCR transgenic mice （Class II-deficient (class II-) mice, Physiological model）,Peripheral blood T cells. | Flow Cytometry | Dual TCR T cells are easily produced in class II-deficient (class II-) mice, and the expression of the second receptor can rescue developing T cells when the rearranged TCR cannot promote the positive selection of its self-MHC molecules(dual TCR T cells). | Smiley, S.T,et al., Dual-receptor T cells expressing one self-restricted TCR. Scand J Immunol, 1997. 45(6): p. 726-30. |
| 1998 | C57BL/10 mice（Physiological model）, TCR+/- heterozygous mice（Physiological model）, Mesenteric lymph node T cells, thymus cells. | Flow Cytometry | Thymocytes from wild-type C57BL/10 mice and TCR+/- heterozygous mice co-express Vα2 with Vα11 or Vα8, and about 10% of CD4+ peripheral T cells in C57BL/10 mice were composed of dual Vα, and CD4+Vα3.2+ T cells appeared to express a second Vα chain at a very high frequency (52%).(dual TCR--*ααβ* T cells)... | Elliott, J.I., Selection of dual Valpha T cells. European journal of immunology, 1998. 28 7: p. 2115-23. |
| 1999 | Transgenic mice（DO11.10 TCR transgenic mice, Physiological model），lamina propria lymphocytes, Peyer’s patches, spleen CD4+ T cells. | Flow Cytometry，immunohistochemistry. | The presence of dual TCR cells specific for intestinal antigens, and the recognition of intestinal antigens by these cells promoted the development and regeneration of memory/effector cell populations that can produce IL-10/IFN-γ.(dual TCR T cells). | Saparov, A., et al., Memory/effector T cells in TCR transgenic mice develop via recognition of enteric antigens by a second, endogenous TCR. Int Immunol, 1999. 11(8): p. 1253-64. |
| 1999 | C57Bl/6 (H-2Db) or DBA/2 (H-2Dd)（H-Y TCR transgenic mice, Physiological model）, Thymus , lymph node T cells. | Flow Cytometry | The positive selection efficiency of dual TCR thymocytes was lower than that of single TCR thymocytes，and the presence of dual TCR T cells may have an effect on the maintenance of self-tolerance.(dual TCR T cells).. | Paterson, R.K., et al., Development and function of autospecific dual TCR+ T lymphocytes. Int Immunol, 1999. 11(1): p. 113-9. |
| 2001 | Transgenic mice（TCR transgenic mice, Physiological model）, thymus cells. | Proliferation test and intracellular FACS analysis | The mechanism of functional allelic exclusive rearrangement in dual TCR transgenic mice is achieved by controlling the TCR assembly process, rather than feedback at the transcription and expression levels..(dual TCR T cells) | Sant'Angelo, D.B., et al., Maintenance of TCR clonality in T cells expressing genes for two TCR heterodimers. Proc Natl Acad Sci U S A, 2001. 98(12): p. 6824-9. |
| 2002 | B10.S mice（KB TCR mice, Physiological model）, thymus, lymph nodes, spleen T cells. | Sanger sequencing, CFSE analysis. | Dual TCR T cells in the thymus/lymph nodes/spleen can effectively respond to foreign antigens presented by their self-MHC molecules, and have the potential to compete with single TCRT cells. Such dual TCR T cells can expand the functional TCR repertoire(dual TCR T cells). | He, X., et al., Dual receptor T cells extend the immune repertoire for foreign antigens. Nat Immunol, 2002. 3(2): p. 127-34. |
| 2003 | Transgenic mice（5CC7/D011.10 rag2-/- TCR transgenic mice and P14/OT-1 TCR transgenic mice, Physiological model）, lymph node T cells and spleen T cells. | Flow Cytometry | All CD4+T cells of the 5CC7/DO11.10 cell line expressed both receptor. Compared with cell lines made from the single TCR-transgenic parental mice, the DO11.10 receptor on the dual-TCR cell line was expressed at relatively high levels (within 2-fold of the DO11.10 single-TCR cell line), whereas the 5CC7 receptor on the dual-TCR line was expressed at 10–15% of the level of the single-TCR 5CC7 line. Compared with cell lines made from the single-TCR-transgenic mice, the cell line generated from the F2 back-cross generation expressed ~30% of the P14 levels and almost equivalent levels of the OT-1 receptor as determined by their expression of Vβ8 and Vβ5, respectively.(dual TCR T cells) | Yang W, Grey HM. Study of the mechanism of TCR antagonism using dual-TCR-expressing T cells. J Immunol. 2003 May 1;170(9):4532-8. |
| 2004 | B6 mice（TCRαβ transgenic mice, Physiological model）， thymus, spleen cells. | Flow Cytometry, T cell proliferation test. | The TCR of many thymic T cells rearranged and expressed two different TCRα chains in TCR transgenic mice, and the number of such cells expressing dual TCR was significantly lower in mature T cells.(dual TCR--*ααβ* T cells).. | Lacorazza, H.D,et al., Exclusion and inclusion of TCR alpha proteins during T cell development in TCR-transgenic and normal mice. J Immunol, 2004. 173(9): p. 5591-600. |
| 2005 | Transgenic mice（AND/ LCMV TCR transgenic mice, Physiological model）, spleen and lymph node T cells. | T cell proliferation assay, Cytotoxicity Assay, Flow Cytometry. | Dual TCR T cells can become tolerant after specific treatment, i.e., no longer produce immune response to antigens that would otherwise activate them. In addition, these dual TCR T cells can also transmit signals through another T cell receptor..(dual TCR T cells) | Hah, C., et al., Induction of peripheral tolerance in dual TCR T cells: an evidence for non-dominant signaling by one TCR. J Biochem Mol Biol, 2005. 38(3): p. 334-42. |
| 2006 | Thymic tissue of healthy children, Peripheral blood mononuclear cell of healthy adults. | Flow Cytometry, Real-time PCR, TCR repertoire analysis. | CD4+ CD25+ T (Treg) cells in peripheral blood of healthy adults expressed dual different Vα chain, estimating that 50% to 99% of CD25+ Treg cells had dual specificity, while CD25- cells were about 20%, and cells with dual TCR also expressed more FOXP3 than cells with a single TCR..(dual TCR--*ααβ* Treg cells).. | Tuovinen, H., et al., Most human thymic and peripheral-blood CD4+ CD25+ regulatory T cells express 2 T-cell receptors. Blood, 2006. 108(13): p. 4063-70. |
| 2007 | CMV seropositive Population，Peripheral blood mononuclear cell specific T cells. | FACS, Retrovirus transduction of T lymphocytes. | The efficiency of TCR cell-surface expression is controlled by the intrinsic quality of the TCR complex. The affinity of dual receptor T cells for their antigen not only depends on TCR affinity, but also on the number of specific TCRs on the cell surface.(dual TCR T cells) | Heemskerk, M.H., et al., Efficiency of T-cell receptor expression in dual-specific T cells is controlled by the intrinsic qualities of the TCR chains within the TCR-CD3 complex. Blood, 2007. 109(1): p. 235-43. |
| 2011 | Lung tissue of mice infected with influenza virus | Multiplex-nested PCR | About 10% of CD8+T cells had two highly efficient TCRα mRNA transcripts..(dual TCR--*αββ* CD8+T cells) | Dash, P., et al., Paired analysis of TCRα and TCRβ chains at the single-cell level in mice. J Clin Invest, 2011. 121(1): p. 288-95. |
| 2016 | Peripheral blood of subjects who cleared hepatitis C virus infection | Reconstruct the native TCRαβ from single cell RNA-seq data of Ag-specific T cells. | They successfully reconstituted functional TCRαβ (89%) in 56 of a total of 63 cells, with dual alpha and dual beta expressed in 18% and 7%, respectively..(dual TCR T cells) | Eltahla, A.A., et al., Linking the T cell receptor to the single cell transcriptome in antigen-specific human T cells. Immunol Cell Biol, 2016. 94(6): p. 604-11. |
| 2016 | C57BL/6N mice（Physiological model）, spleen CD4+T cells. | TraCeR calculation method | 42% of cells detected dual α chain in C57BL/6N mouse spleen CD4+ T cells, dual β chain in 22% of cells, dual functional α chain in 19% of cells, and dual functional β chain in 6% of cells, which is far higher than the proportion detected by various methods before.(dual TCR T cells) | Stubbington, M.J.T., et al., T cell fate and clonality inference from single-cell transcriptomes. Nat Methods, 2016. 13(4): p. 329-332. |
| 2020 | Artificially constructed B6.TCRA-GFP/RFP transgenic mice | Flow Cytometry; Confocal Microscope; pMHC tetramer. | In naive mice, the proportion of double TCR alpha cells in CD4+and CD8+T cells is about 16% (much higher than traditional cognition of 1-10%), and the proportion is consistent with confocal microscopy verification (16.0 ± 1.0%) .(dual TCR--*αββ* CD8+T cells) | Yang L, Jama B, et al., TCRα reporter mice reveal contribution of dual TCRα expression to T cell repertoire and function. Proc Natl Acad Sci USA 2020;117:32574–83. |
| 2022 | Tuberculosis patients, healthy children, adults and elderly, peripheral blood mononuclear cell and donor spleen | Flow Cytometry，Illumina high-throughput sequencing, single cell TCR pairing sequencing | Mucosal Associated Invariant T (MAIT), in addition to expressing the classical TCRα chain (TRAV1-2), can also express both atypical TCRα chains, and when these MAIT cells bind to the specific antigen (5-OP-RU), They can generate specific responses through these classical and atypical TCRα chains..(dual TCR--*α*αβ MAIT cells) | Suliman, S., et al., Dual TCR-α Expression on Mucosal-Associated Invariant T Cells as a Potential Confounder of TCR Interpretation. J Immunol, 2022. 208(6): p. 1389-1395. |
| 2023 | Human thymus and peripheral blood, C57BL/6 mice (Physiological model) lymph nodes and spleen and peripheral blood T cells . | scRNA+TCR-seq, Flow cytometry. | The presence of single T cells expressing multiple TCR chains in all samples, with the proportion of 15%, 10%, and 20% in the human thymus, human peripheral blood, and mouse lymphoid organs, respectively. Multiple TCR one of TCR β (orTCR α) should originate from the transcription of V(D)J combination in T-cell receptor excision circle (TREC) formed after the twice successful rearrangement in the same chromosome. Moreover, human V30 (or mouse V31) gene may participate in reverse recombination and transcription to prevent allelic exclusion.(dual TCR T cells). | Zhu, L., et al., scRNA-seq revealed the special TCR β & α V(D)J allelic inclusion rearrangement and the high proportion dual (or more) TCR-expressing cells. Cell Death Dis, 2023. 14(7): p. 487. |
| 2023 | Human (fetus and newborns, young individual, old individual), cord blood, thymic. | scRNA -seq | Multiple TCR T cells were least prevalent in young individuals, and the proportion was significantly lower than that in the elderly population. Age-related thymic involution may be responsible for the high proportion of multiple TCR T cells among the elderly..(dual TCR T cells). | Jun L,et al., A new immunological index for the elderly: high proportion of multiple TCR T cells based on scRNA-seq. Aging Dis. 2023 Sep 4. doi: 10.14336/AD.2023.0509. Epub ahead of print. PMID: 37733445. |
| 2025 | Mouse lymphoid tissue and non lymphoid tissue | scRNA+TCR-seq | There are a certain proportion of double TCR Treg cells in different tissue parts of mice, and their VDJ family is used. The mRNA expression of functional molecules varies, suggesting that their complex regulatory effects may be related to Treg in different tissues.(dual TCR Treg cells) | [Yuanyuan Xu](https://elifesciences.org/reviewed-preprints/105504#x2124501189)，et al., scRNA+TCR-seq Reveals the Proportion and Characteristics of Dual TCR Treg Cells in Mouse Lymphoid and on-lymphoid Tissues. Elife. 2025, [https:// doi.org/ 10.7554/eLife.105504.2](https://doi.org/10.7554/eLife.105504.2) |
| 2025 | The scRNA+VDJ seq data in the shared database consists of 147 samples. | Develop DeRR analysis tool to screen for dual TCR in single-cell TCR and RNA sequencing data. | 147 samples, 17% double TCR alpha chains, 12% double TCR beta chains; Increased dual TCR frequency in cancer; There is a positive correlation between autoimmune diseases and disease duration..(dual TCR Treg cells) | Si-Yi Chen , et al., DeRR: A Unique Detecting Method and the First Landscape for T Cells with Dual T Cell Receptors from Large-scale Single Cell Data  Genomics Proteomics Bioinformatics'. 2025 Oct 6:qzaf090. |

**Sup Table 2-2. In pathological conditions of human and mouse** (tumor; autoimmune disease; infectious diseases; transplantation immunity, etc.). The main overview and progress of the research objects, methods, and characteristics of dual TCR T cells.

| Time | Study object, sample | Detection methods | Proportion, function, composition characteristics, etc | References |
| --- | --- | --- | --- | --- |
| 1988 | Human diphtheria toxoid specific CD4+T cells(infectious diseases). | Sanger sequencing,Southern blot | Use two functional Vβ family 1.3 kb β transcripts, Vβ3-Dβ1-1-J β 1-1-Cβ1, and Vβ4-Dβ1-Jβ 2-1-Cβ2, The rule of allele exclusion is not followed(dual TCR--*αββ* T cells).. | Triebel, F., et al., Evidence for expression of two distinct T cell receptor beta-chain transcripts in a human diphtheria toxoid-specific T cell clone. J Immunol, 1988. 140(1): p. 300-4. |
| 1991 | Plasmodium berghei circumsporozoite immunized mice，spleen T cells(infectious diseases).. | Flow Cytometry，Sanger sequencing. | At least 8 (29%) of 28 peripheral CTL clones from the spleens of mice immunized with Plasmodium berghei circumsporozoite expressed two functional TCRα transcriptse(dual TCR--*ααβ* T cells)... | Casanova, J.L., et al., T cell receptor genes in a series of class I major histocompatibility complex-restricted cytotoxic T lymphocyte clones specific for a Plasmodium berghei nonapeptide: implications for T cell allelic exclusion and antigen-specific repertoire. J Exp Med, 1991. 174(6): p. 1371-83. |
| 1995 | TCR α-hemizygous mice（Experimental allergic encephalomyelitis, lupus and NOD mice pathological model）（Autoimmune diseases）. | Genotypic Analysis，Flow Cytometry，T Cell Proliferation Assays，Disease Induction and Analysis. | The incidence of experimental allergic encephalomyelitis and lupus is not affected by dual TCRα cell deficiency, and nonobese diabetic (NOD) TCRα hemizygote has a significant protective effect against cyclophosphamid-accelerated islet and diabetes (dual TCR--*ααβ* T cells). | Elliott, J.,et al., Dual T cell receptor alpha chain T cells in autoimmunity. J Exp Med, 1995. 182(4): p. 953-9. |
| 1995 | Normal donors, peripheral blood mononuclear cells（Autoimmune diseases）.. | Flow Cytometry | About 1/3 of peripheral T cells in healthy people express two Vα Chains (Va24 and Va2 or Va12).(dual TCR--*ααβ* T cells). | Padovan, E., et al., Dual receptor T-cells. Implications for alloreactivity and autoimmunity. Ann N Y Acad Sci, 1995. 756: p. 66-70. |
| 1998 | BALB/c mice（Physiological model）, splenocytes.（Autoimmune diseases）.. | Flow Cytometry, immunohistochemistry. | One of the reasons T cells evade self-tolerance is the expression of dual TCR, which can occur in 30% of T cells in normal mice, which can lead to a low-level of expression of autospecific TCR T cells.(dual TCR--*ααβ* T cells)... | Sarukhan, A., et al., Allelic inclusion of T cell receptor alpha genes poses an autoimmune hazard due to low-level expression of autospecific receptors. Immunity, 1998. 8(5): p. 563-70. |
| 1999 | Hybrid mice（BDC2.5 transgenic mice with the A18 transgenic strain, NOD pathological model）.（Autoimmune diseases） | Flow Cytometry | The triggering of a second TCR expressed on diabetogenic T cells may lead to the onset of diabetes..(dual TCR T cells) | Fossati, G., et al., Triggering a second T cell receptor on diabetogenic T cells can prevent induction of diabetes. J Exp Med, 1999. 190(4): p. 577-83. |
| 2001 | B10.Q mice（TCRα +/- hemizygous mice, Physiological model）, wild-type mice, peripheral blood T cells.（Autoimmune diseases） | Flow Cytometry | In wild-type mice and hemizygous (Tcr α+/-) mice, about 8% of T cells in peripheral blood express dual TCR α chains..(dual TCR T cells) | Corthay, A., K.S,et al., Evaluation of the percentage of peripheral T cells with two different T cell receptor alpha-chains and of their potential role in autoimmunity. J Autoimmun, 2001. 16(4): p. 423-9. |
| 2004 | P14/Rag1–/– mice（Vα2 and Vβ8.1 TCR, Physiological model）, OT-I/Rag1^–/–^ mice（Vα2 and Vβ5.1 transgenic TCR, Physiological model）, spleen T cells.(tumor) | Flow Cytometry，CFSE T cell proliferation assay. | Specific dual TCR T cells can be stimulated by two different antigenic peptides to proliferate and produce interferon-γ (IFN-γ), these T cells simultaneously express two different TCRs, one sensitive to ovalbumin (ova257) and the other sensitive to lymphocytic choriomeningitis virus glycoprotein (gp33). Both TCRs mediate anti-tumor responses during the proliferation and production of interferon-γ in these dual TCR T cells..(dual TCR T cells) | Gladow, M., et al., Dual T cell receptor T cells with two defined specificities mediate tumor suppression via both receptors. Eur J Immunol, 2004. 34(7): p. 1882-91. |
| 2004 | SCID-bg mice（Pathological model）,spleen and mesenteric lymph node T cells.(infectious diseases).. | Flow Cytometry, proliferation assay. | Most dual TCR cells were present in the activated/memory T cell and Treg cell subsets..(dual TCR T cells) | Zhou, P., et al., Expression of dual TCR on DO11.10 T cells allows for ovalbumin-induced oral tolerance to prevent T cell-mediated colitis directed against unrelated enteric bacterial antigens. J Immunol, 2004. 172(3): p. 1515-23. |
| 2004 | BALB/c mice（TCR transgenic DO-11.10 mice recognizing ovalbumin peptide 323-33914 and TCR transgenic influenza A hemagglutinin mice, Physiological model）, spleen and lymph node CD4+T cells, Lymph nodes and lung tissue.(infectious diseases) | Intracellular cytokine staining, Lung sensitization experiment. | Different cytokine secretion and effect manifestations in the dual receptor T cell populations of virus-specific TH1 or TH2 cells..(dual TCR T cells) | Aronica, M.A., et al., Susceptibility to allergic lung disease regulated by recall responses of dual-receptor memory T cells. J Allergy Clin Immunol, 2004. 114(6): p. 1441-8. |
| 2007 | Transgenic mice（OT-I/Rag1-/- TCRVα2Vβ5 transgenic mice and 14/Rag1-/-TCRVα2Vβ8 mice, Pathological model）, spleen cells and fibrosarcoma cells.(tumor) | Flow Cytometry | Adoptively transferred dual TCR T cells can be safe and effective, even if the antitumor TCR is expressed at reduced level, provided the tumor  Ag is expressed at sufficient level and the self-Ag at low level..(dual TCR CD8+ T cells) | Weinhold, M., et al., Dual T cell receptor expressing CD8+ T cells with tumor- and self-specificity can inhibit tumor growth without causing severe autoimmunity. J Immunol, 2007. 179(8): p. 5534-42. |
| 2009 | GVHD model mice（Pathological model）, spleen and lymph node T cells.(transplantation immunity) | Flow Cytometry | Spleen and lymph node dual TCR T cells accounted for 60% of peripherally activated T cells in graft-versus-host disease (GVHD) model mice, compared to 1-6% in control mice. Dual TCR T cells from GVHD mice were more likely to be activated.(dual TCR T cells) | Morris, G.P,et al., Cutting edge: Highly alloreactive dual TCR T cells play a dominant role in graft-versus-host disease. J Immunol, 2009. 182(11): p. 6639-43. |
| 2010 | Transgenic mice（Vβ14 and Vβ8.2 transgenic mice, Physiological model）, Thymus and spleen..(transplantation immunity) | Flow Cytometry | Both alleles in-frame V-D-J-Cβ rearrangements in 1–10% of T cell in the thymus and spleen in Vβ14 and Vβ8.2 transgenic mice..(dual TCR--*αββ* T cells).. | Steinel, N.C., et al., Posttranscriptional silencing of VbetaDJbetaCbeta genes contributes to TCRbeta allelic exclusion in mammalian lymphocytes. J Immunol, 2010. 185(2): p. 1055-62. |
| 2010 | Transgenic mice（MHC class II-deficient MBP-specific TCR Vα8 Vβ8 (8.8) transgenic mice, Infection with wild-type vaccinia virus）(infectious diseases).（Autoimmune diseases） | Flow Cytometry | Virus-activated T cells expressed dual TCR, which could recognize MBP and viral antigens, and may be related to infection induced autoimmunity..(dual TCR CD8+T cells) | Ji, Q., A. Perchellet,et al., Viral infection triggers central nervous system autoimmunity via activation of CD8+ T cells expressing dual TCRs. Nat Immunol, 2010. 11(7): p. 628-34. |
| 2011 | DO11.10 mice（Physiological model），spleen and lung tissue T cells.(infectious diseases). | Flow Cytometry，T cell proliferation assay, cell adoptive transfer, RT-qPCR. | A CD4+ cell population in DO11.10 mice, in which approximately 15% of cells expressed additional endogenous TCR alpha chain, indicating dual antigen specificity(dual TCR T cells) | Lemaire, M.M., et al., Dual TCR expression biases lung inflammation in DO11.10 transgenic mice and promotes neutrophilia via microbiota-induced Th17 differentiation. J Immunol, 2011. 187(7): p. 3530-7. |
| 2012 | C57BL/6 mice（KRN T-cell receptor transgenic mice, Pathological model）, thymus and spleen CD4+ T cells.（Autoimmune diseases） | Flow Cytometry | Dual TCR-β expression can enhance the maturation of autoreactive pathogenic T cells and lead to the development of autoimmune diseases..(dual TCR--*αββ* T cells) | Auger, J.L., et al., Incomplete TCR-β allelic exclusion accelerates spontaneous autoimmune arthritis in K/BxN TCR transgenic mice. Eur J Immunol, 2012. 42(9): p. 2354-62. |
| 2013 | Patients receiving allogeneic or autologous hematopoietic stem cell transplantation，Healthy Volunteers, peripheral blood lymphocytes.(transplantation immunity) | Flow Cytometry，ELISA. | Clone specific dual TCR T cells from symptomatic GVHD patients exhibited pathological allogeneic responses to donor mismatched HLAs. Dual TCR T cells appeared 5.3 times more frequently in symptomatic acute GVHD patients than healthy.(dual TCR T cells) | Morris, G.P., et al., Dual receptor T cells mediate pathologic alloreactivity in patients with acute graft-versus-host disease. Sci Transl Med, 2013. 5(188): p. 188ra74. |
| 2014 | Hemizygous mice（TCRα+/- mice, Physiological model）, thymus and spleen CD4+ T cells.(transplantation immunity) | Flow Cytometry, Illumina MiSeq, intracellular cytokine staining. | The efficient development of thymus cells requires simultaneous rearrangement of two TCRαgene sites, and the second TCR has an enhanced response ability to itself and allogeneic pMHC..(dual TCR T cells) | Ni, P.P., et al., The ability to rearrange dual TCRs enhances positive selection, leading to increased Allo- and Autoreactive T cell repertoires. J Immunol, 2014. 193(4): p. 1778-86. |
| 2017 | TCR α knockout NOD mice（Pathological model），TCR β knockout NOD mice（Pathological model）, Thymus and pancreatic drainage lymph nodes（Autoimmune diseases） | Flow Cytometry | Dual TCR T cells change the composition of autoreactive T cell populations by restricting autoreactive thymus cells to Treg cell lineage selection , resulting in a phenotype with lower tolerance and more prone to autoimmunity..(dual TCR T cells) | Schuldt, N.J., et al., Cutting Edge: Dual TCRα Expression Poses an Autoimmune Hazard by Limiting Regulatory T Cell Generation. J Immunol, 2017. 199(1): p. 33-38. |
| 2017 | Healthy volunteers and hematopoietic stem cell transplant patients, Peripheral blood T cells.(transplantation immunity) | Flow Cytometry，Single-cell RNA sequencing | The number of dual TCR expressing lymphocytes increased in haematopoietic stem cell transplant( HSCT) patients with GVHD..(dual TCR T cells) | Balakrishnan, A., et al., Proinflammatory Dual Receptor T Cells in Chronic Graft-versus-Host Disease. Biol Blood Marrow Transplant, 2017. 23(11): p. 1852-1860. |
| 2022 | C57BL/6 mice (VSV and EGFRvIII dual-specific CAR – T, Pathological model), spleens, blood, tumors, skin cells..(infectious diseases). | Flow Cytometry | Stimulation of the native T cell receptor (TCR) with viral or virally encoded epitopes gives rise to enhanced proliferation, CAR-directed antitumor function, and distinct memory phenotypes. This treatment led to prolonged survival of mice with subcutaneous melanoma and intracranial glioma tumors.(dual-specific CAR T cells ) | Evgin, L., et al., Oncolytic virus-mediated expansion of dual-specific CAR T cells improves efficacy against solid tumors in mice. Sci Transl Med, 2022. 14(640): p. eabn2231. |
| 2023 | Aransgenic mice (B6.TCRα(TCRA)-green fluorescent protein (GFP)/red fluorescent protein (RFP) transgenic mouse, Physiological model), B57BL/6 mice (6727 sarcoma tumors), spleen, tumor mass, contralateral and draining lymph nodes cells..(tumor) | Flow Cytometry | Dual TCR cells were specifically increased among tumor-infiltrating lymphocytes (TILs) in both models, indicating selective advantage in antitumor responses. Phenotype and single-cell gene expression analyses identified dual TCR are predominant during the effective antitumor response, demonstrating selectively increased activation in the TIL compartment and skewing toward an effector memory phenotype..(dual TCR T cells) | Jang, H.J., et al., Dual receptor T cells mediate effective antitumor immune responses via increased recognition of tumor antigens. J Immunother Cancer, 2023. 11(5). |
| 2024 | Human, blood, synovial fluid（Autoimmune diseases） | scRNA+TCR-seq | More than 10% dual TCR pTh17 and dual TCR Treg T cells involvement in autoimmune response in ankylosing spondylitis., May play an important role in the occurrence and development of AS. .(dual TCR pTh17 and Treg cells). | Yuanyuan Xu,et al., scRNA+TCR-seq revealed the dual TCR pTh17 and Treg T cells involvement in autoimmune response in ankylosing spondylitis. International Immunopharmacology. 135 ,2024, 12279. 3. |
| 2024 | Human, blood, tumor tissue, Peritumoral tissue..(tumor) | scRNA+TCR-seq | More than 10% dual TCR T cells antitumor response in the Tumor microenvironment of non-small cell lung cancer. Presenting varying degrees of clonal proliferation, confirming its involvement in anti-tumor response effects..(dual TCR T cells). | Peng Q, et al., scRNA+ TCR-seq revealed dual TCR T cells antitumor response in the TME of NSCLC. Journal for ImmunoTherapy of Cancer 2024;12:e009376. |
| 2024 | Human,blood.（Autoimmune diseases） | scRNA+TCR-seq | About 10% of dual receptor T cells may be involved in the occurrence and development of Kawasaki disease, and are closely related to the response to immunoglobulin therapy(dual TCR T cells). | Xu Y,et al., [scRNA+TCR-seq reveals the pivotal role of dual receptor T lymphocytes in the pathogenesis of Kawasaki disease and during IVIG treatment.](https://pubmed.ncbi.nlm.nih.gov/39421738/) Front Immunol. 2024 Oct 3;15:1457687. |
| 2025 | TLS tissue in pemphigus vulgaris；Healthy volunteer skin tissue.（Autoimmune diseases） | scRNA+TCR-seq | There is a certain proportion of dual TCR T cells involved in the pathological process of pemphigus vulgaris in TLS tissue, and dual TCR CXCL13+CD4T cells in TLS tissue of pemphigus vulgaris are involved in disease progression..(dual TCR Treg cells) | Quan K, Yet al., Single-Cell RNA Sequencing + TCR Sequencing Reveal the Proportion and Characteristics of Dual TCR T Cells in Tertiary Lymphoid Structures in Pemphigus. J Invest Dermatol. 2025 May 24:S0022-202X(25)00524-X. |
